# Lectin affinity chromatography and quantitative proteomic analysis reveal that galectin-3 is associated with metastasis in nasopharyngeal carcinoma

**DOI:** 10.1038/s41598-020-73498-y

**Published:** 2020-10-05

**Authors:** Sathid Aimjongjun, Onrapak Reamtong, Tavan Janvilisri

**Affiliations:** 1 Graduate Program in Molecular Medicine, Faculty of Science, Mahidol University, Bangkok, 10400 Thailand; 2 Department of Biochemistry, Faculty of Science, Mahidol University, Bangkok, 10400 Thailand; 3 Department of Molecular Tropical Medicine and Genetics, Faculty of Tropical Medicine, Mahidol University, Bangkok, 10400 Thailand

**Keywords:** Tumour biomarkers, Proteomics

## Abstract

Nasopharyngeal carcinoma (NPC) is a serious cancer in East and Southeast Asia. Patients are often diagnosed at advanced stages, rendering treatment failure due to high potential of metastasis. This study identified lectin-binding glycoproteins with a potential role in NPC metastasis. Cell lysate and culture medium in highly metastatic 5-8F, and lowly-metastatic 6-10B NPC cell lines were fractionated by ConA- and WGA-affinity chromatography, and subjected to GeLC-MS/MS. A total of 232 and 197 proteins were identified in ConA-enriched fraction of 5-8F and 6-10B cell lysates respectively. In WGA-enriched fraction, 65 and 164 proteins were found in 5-8F and 6-10B cell lysates respectively. Proteins identified in culture medium for both cell lines were 223 and 85 for ConA-enriched fraction, and 94 and 124 for WGA-enriched fraction from 5-8F and 6-10B respectively. Differentially expressed proteins were functionally categorized into cell–cell adhesion, extracellular matrix, glycolysis, protein homeostasis and/or glycosylation enzymes, and lipid metabolism. Interestingly, Galectin-3 (Gal-3) was highly expressed in 5-8F cells but was lowly expressed in 6-10B cells. The Gal-3 knockdown in 5-8F cells, Gal-3 overexpression in 6-10B cells and treatment with Gal-3 inhibitor revealed that Gal-3 was responsible for metastatic phenotypes including adhesion, migration and invasion. So Galectin-3 may serve as a potential target for NPC therapeutic interventions.

## Introduction

Nasopharyngeal carcinoma (NPC) is an Epstein-Barr virus-associated malignant cancer that originates from the epithelial cells of the nasopharynx. A high incidence is found in China and the Southeast Asia including Thailand^1^. Most NPC cases present with advanced stages due to the late diagnosis, hence NPC usually exhibits higher metastatic potential than other head and neck carcinoma^2^. Although, radiotherapy represents standard treatment for NPC, the patients with advanced of NPC have poor response, and locoregional recurrences and distant metastases have been observed. Chemotherapy is usually introduced concurrently with radiotherapy to ameliorate the survival for advanced NPC, however the responses from patients with recurrent or metastatic NPC have not been successful^3^. Thus, NPC has an extremely poor prognosis, urging us to understand the molecular mechanisms of NPC pathogenesis and progression, to find suitable biomarkers for early detection, and to search for therapeutic targets^4,5^. Unfortunately, the knowledge of NPC pathogenesis, including molecular mechanisms of recurrent or metastatic of NPC is very limited.

Post-translational modifications (PTM) pose as an important mechanism to modulate cellular proteomes, which are critical for a wide range of biological functions such as cell differentiation, degradation of proteins, protein signaling, control of gene expression, and protein–protein interactions^6^. Among the many types of PTM, glycosylation have been found in a variety type in cancers and appear to be the complex. Glycoproteins has gained attention as they have been associated with carcinogenesis, progression and metastasis in many types of cancer^7^ and have been used as biomarkers in the clinic including alpha-fetoprotein, CA125, CEA, CA19-9 and PSA^8^. Hence, understanding of the proteomes and its influence on disease progression drives the demand for extensive identification of aberrant glycoproteins under different cellular or disease states. At present, proteomic data on NPC biomarkers revealed proteins linked to cancer progression involved in cell movement, cell cycling, transcription, regulation and apoptosis. These include cathepsin B, cathepsin C, cofilin-1, profilin-1, L-lactate dehydrogenase A chain, 14-3-3σ, heat shock cognate 71 kDa, and stathmin, which has been identified in secretory proteins of NPC cells^9^. Differentially expressed proteins involved in metastatic process including peroxiredoxin 3, peroxiredoxin 6, superoxide dismutase, prohibitin Nm-23-H1, 14-3-3σ, HSP 27, maspin, heterogeneous nuclear ribonucleoproteins C1/C2, α-enolase, and annexin-A1 and triosephosphate isomerase have been identified^10,11^. However, mining for glycosylated protein biomarkers in NPC has not been investigated.

In the present study, we attempted to identify lectin-specific glycoproteins which potentially play a role in NPC metastasis through lectin affinity chromatography and quantitative proteomic analysis. As lectins are proteins that can specifically and reversibly bind carbohydrates, hence subjecting proteins to lectin affinity chromatography enables enrichment of different classes of glycoproteins. Concanavalin A (Con A) lectins can capture peptides with high-mannose type N-glycan, while wheat germ agglutinin (WGA) can capture N-Acetyl glucosamine (GlcNAc) and interact to sialic acid containing glycoconjugates and oligosaccharides^12^. Both ConA- and WGA-enriched fractions from cell lysates and culture media of highly and lowly metastatic NPC cell lines were subjected to liquid chromatography mass spectrometry. Here, Galectin-3 has been identified to play a role in NPC metastasis. Our data provide new insights to understanding and identify aggressive biomarkers that may accelerate cancer metastasis in NPC cells, which may serve as potential therapeutic targets for NPC.

## Results

### Phenotypic characterization of highly and lowly metastatic NPC cells

**Figure 1:**
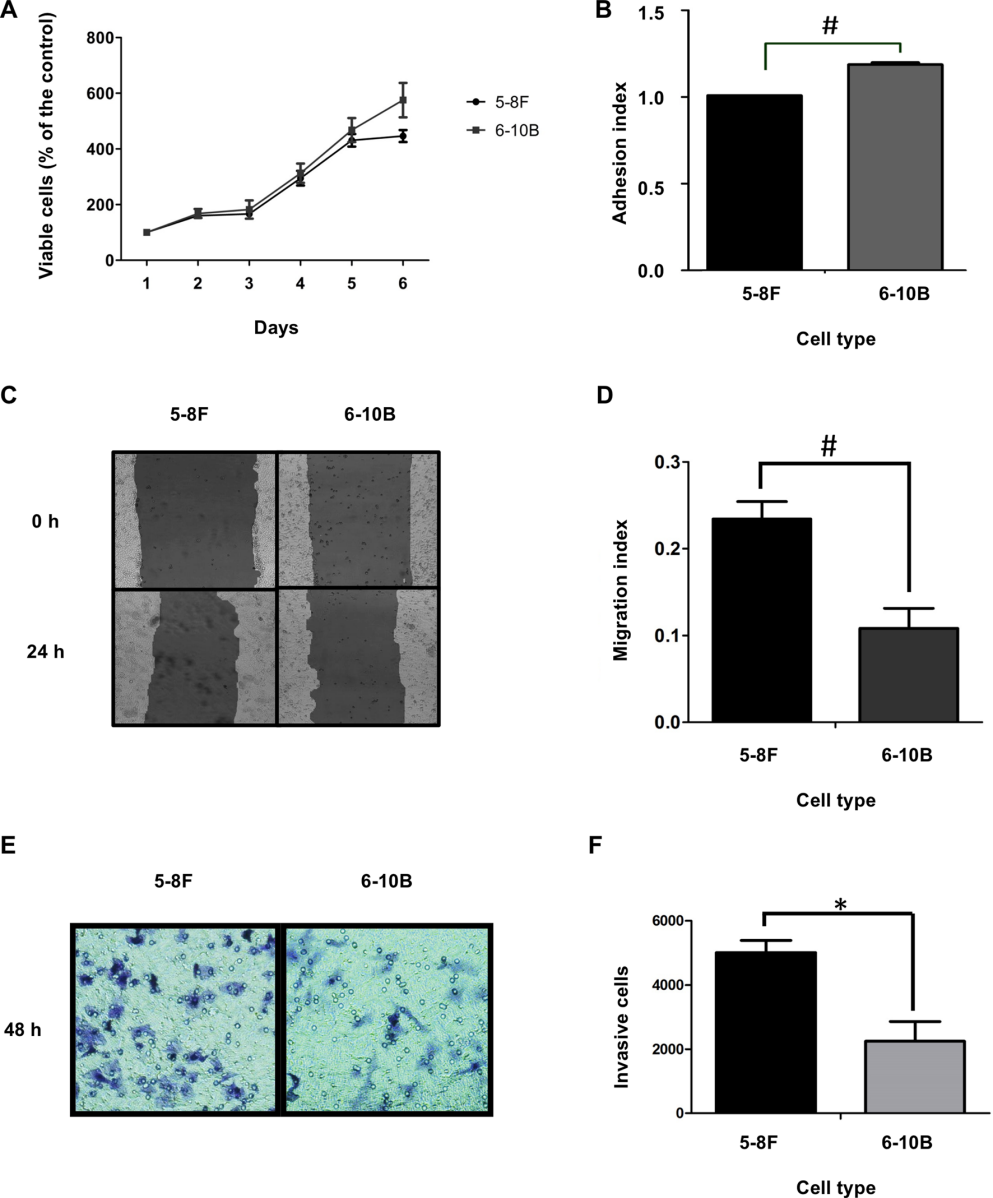
Phenotypic characterization of 5-8F and 6-10B NPC cells. (**A**) The growth rate of 5-8F and 6-10B cells was determined by MTT assay. (**B**) In vitro results of adhesion assay on 5-8F and 6-10B cells. (**C**) Representative photographs of scratch wound healing assay with (**D**) the migration index between 5-8F cell and 6-10B cells. (**E**) Representative photographs of in vitro Transwell assay with (F) the quantitation of invasive 5-8F and 6-10B cells. Each bar represents the mean ± SEM *, *P* < 0.05 and # < 0.001.

In our study, we used two NPC cell lines including highly tumorigenic and metastatic (5-8F) and highly tumorigenic but not metastatic (6-10B) NPC cells, both of which were derived from the same parental cell line. The results revealed that the growth rates of both cells were similar (Fig. 1A), however, differential metastatic potentials of 5-8F and 6-10B were observed. Based on our data, 5-8F cells exhibited lower adhesive behaviors to adhere to the extracellular matrix (ECM) (Fig. 1B), but higher migrative and invasive capabilities compared to 6-10B cells. (Fig. 1C–F).

### Differential glycoproteins in highly and lowly metastatic NPC cells

**Figure 2:**
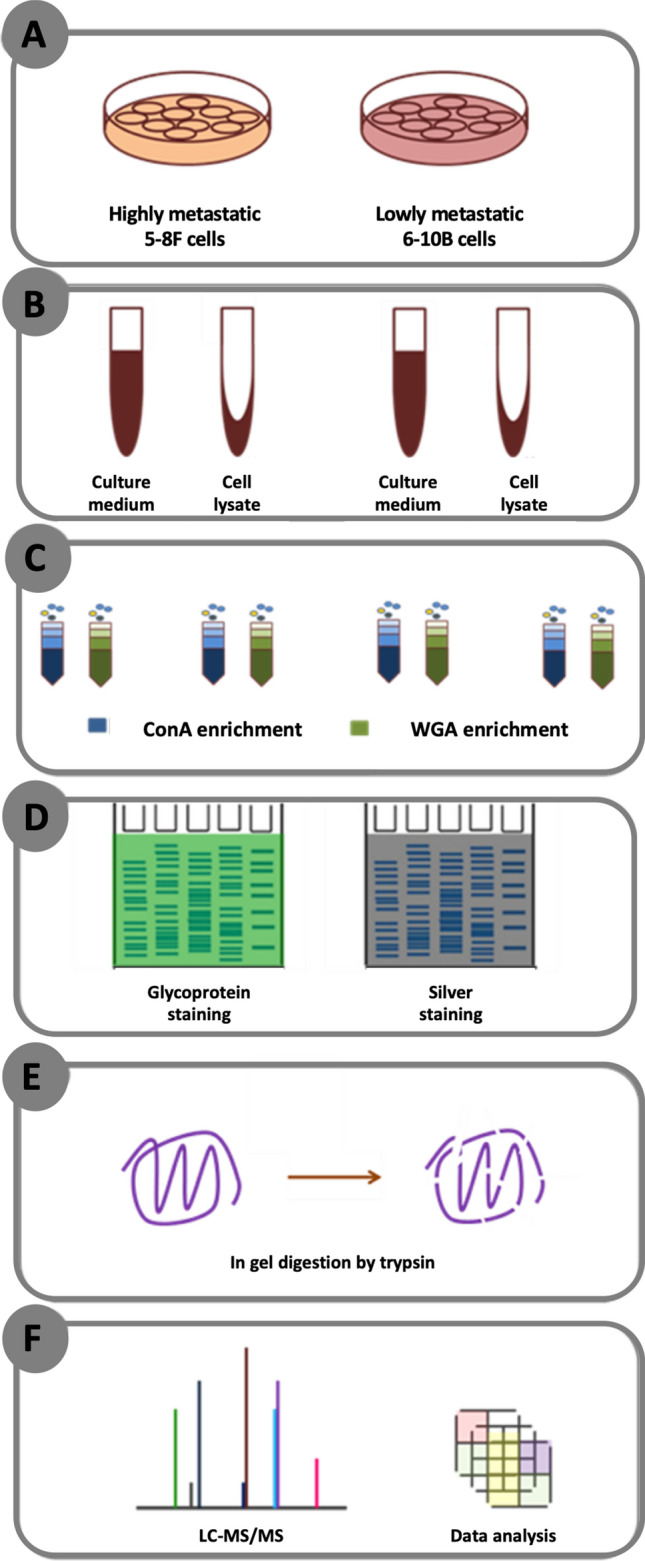
Schematic representation of the experimental workflow. (**A**) Differentially metastatic 5-8F and 6-10B NPC cells were cultured. (**B**,**C**) The proteins in cell lysates and culture medium obtained from both cells were extracted with the enrichment on lectin affinity chromatography enrichment fractions using ConA- and WGA-binding columns. (**D**) Lectin-binding proteins from the enrichment were separated on SDS-PAGE, which were then subjected to glycoprotein and silver staining. (**E**,**F**) The small pieces of cut gel were trypsin digested prior to label free LC–MS/MS and data analysis.

**Figure 3:**
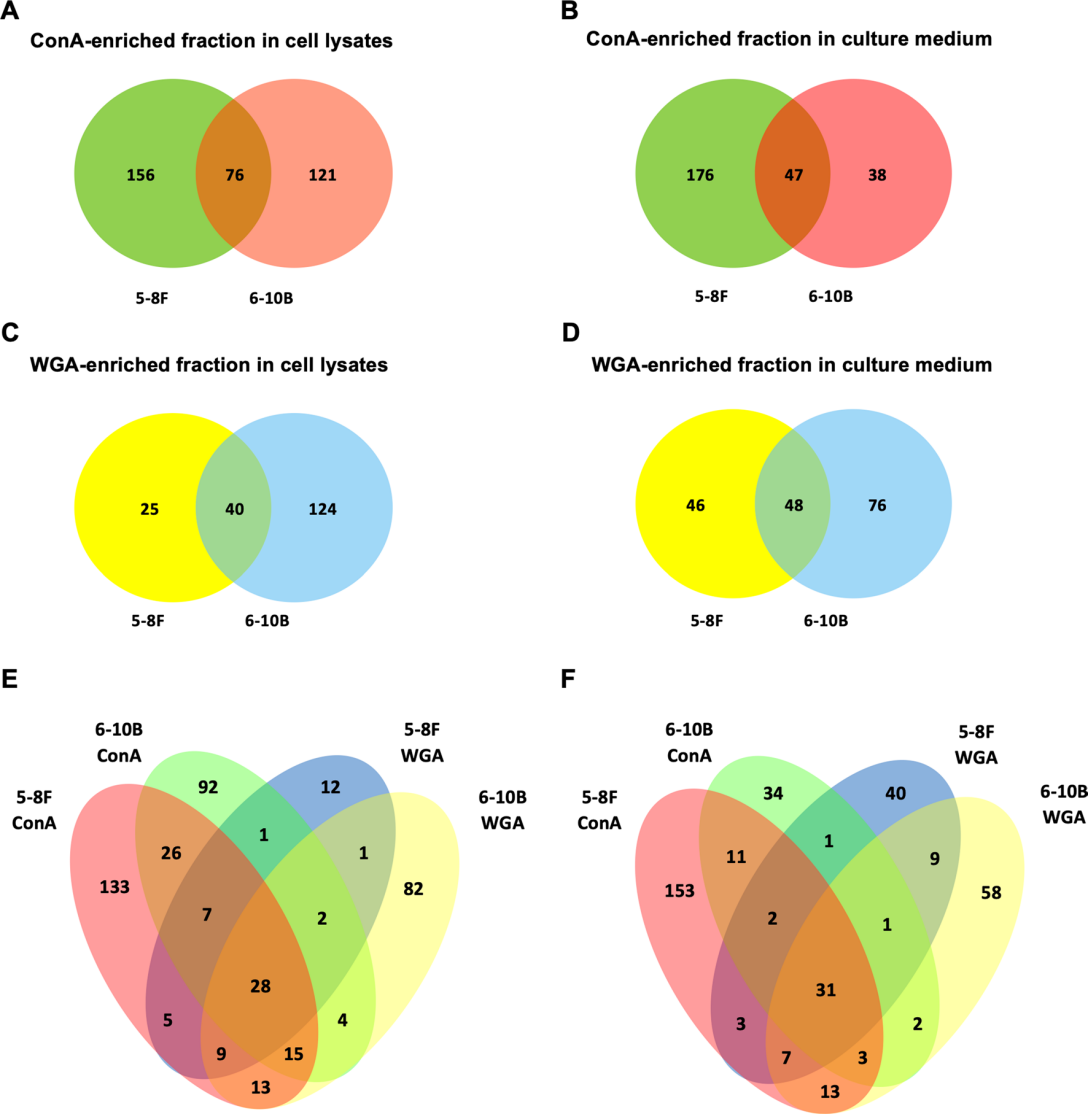
Profiling of the proteins identified by LC–MS/MS. Glycoprotein fractions from differentially metastatic 5-8F and 6-10B NPC cells were captured by ConA and WGA-binding columns and identified by nano LC–MS/MS. Venn diagrams represent the ConA-enriched fraction in (**A**) cell lysates and (**B**) culture medium, and the WGA-enriched fraction in (**C**) cell lysates and (**D**) culture medium in both cells. Comparison of protein identification in both ConA-enriched and WGA-enriched fractions in (**E**) cell lysates and (**F**) culture medium was also performed and represented in Venn diagrams.

The experimental workflow for our study is shown in a schematic representation (Fig. 2). Total proteins from cell lysates and culture media of 5-8F and 6-10B NPC cells were captured in ConA- and WGA-affinity chromatography specific to mannose-rich and sialic acid-rich glycans, respectively. The proteins in the eluted fraction were stained with silver stain and periodic acid-Schiff (PAS), which is specific to glycosylated proteins to ensure the same loading amount. To characterize the proteomes, retrieved fractions were separated by SDS-PAGE and digested by trypsin prior to nano ESI MS/MS analysis. The results showed 353 proteins in ConA-enriched fraction in cell lysates (Fig. 3A) and 261 in culture medium (Fig. 3B). There were 76 and 47 common ConA-enriched proteins present in both 5-8F and 6-10B cell lysates and culture medium respectively. For WGA-enriched fractions, a total of 189 and 170 proteins were identified in cell lysates (Fig. 3C) and culture medium (Fig. 3D) respectively. In addition, the overlapping proteins found in both two cell lines were 40 in cell lysates and 48 in culture medium. In cell lysates, there were 319 (74.2%) proteins unique for ConA-enriched fractions and WGA-enriched fractions in 5-8F and 6-10B cells and only 28 (6.5%) lectin-binding proteins identified in all fractions (Fig. 3E). In culture medium, there were 31/368 (8.4%) common lectin-binding proteins identified in all fractions and 285 (77.4%) unique for each fraction in both cell types (Fig. 3F).

Differentially expressed glycoproteins were clustered using DAVID gene functional classification tool. The analysis of the abundance of each functional category revealed substantial differences in all conditions. Here, we focused on the proteins that were involved in cancer metastasis, especially cell–cell adhesion, extracellular matrix, wound healing and secreted proteins (Tables 1, 2, 3 and 4). There were proteins that play roles in cell–cell adhesion found differentially expressed in 5-8F and 6-10B cells such as CD44, MUC13, transferrin receptor and major histocompatibility, which were highly expressed in 5-8F cells, while integrin alpha 3 and 5 were only expressed in 6-10B cells. For secreted proteins from culture medium, soluble proteins such as s100A8 and s100A9, which have been shown to be associated with cancer cell invasion, were only detected in highly metastatic 5-8F cells, but not lowly-metastatic 6-10B cells. Candidate proteins were further selected by considering proteins that exhibited more than 1.5-fold of protein scores corresponding to the EMPAI values. Interestingly, the expression of galectin-3 was found ~ 2.1-fold higher in 5–8 cells compared to 6-10B cells, while galectin-3 binding protein was found only in WGA-enriched fraction in 5-8F, but not 6-10B cells. Based on evidence for its roles in metastatic potential in cancer cells, the availability of specific inhibitors and no previous reports in NPC, galectin-3 was selected for further validations.

**Table 1:** Peptides in identified in ConA-enriched fraction from 5-8F and 6-10B cell lysates. (**A**) Extracellular matrix-associated lectin-binding proteins involved in metastasis and (**B**) Cell surface- associated lectin-binding proteins involved in metastasis.

ConA lectin-binding cell lysates							
Extracellular matrix						EMPAI	
ID	Acession no.	Protein name	Net N glycosylated	Protein mass	PI	5-8F	6-10B
**A**							
ATPA_HUMAN	P25705	ATP synthase, H + transporting, mitochondrial F1 complex, alpha subunit 1, cardiac muscle(ATP5A1)	40 N, 89 N, 113 N, 384 N, 409 N,	59,714	9.16	0.06	0.11
ATPB_HUMAN	P06576	ATP synthase, H + transporting, mitochondrial F1 complex, beta polypeptide(ATP5B)	84 N, 146 N, 222 N, 244 N, 307 N	56,525	5.26	0.12	N/A
ACTG_HUMAN	P63261	Actin gamma 1(ACTG1)	12 N, 78 N, 111 N, 115 N, 296 N	41,766	5.31	0.7	0.7
ANXA2_HUMAN	P07355	Annexin A2(ANXA2)	32 N, 41 N, 62 N, 258 N	44,524	6.1	N/A	0.09
CATD_HUMAN	P07339	Cathepsin D(CTSD)	73 N, 102 N, 134 N, 217 N, 227 N, 263 N	44,524	6.1	0.24	0.07
COF1_HUMAN	P23528	Cofilin 1(CFL1)	16 N	18,491	8.22	0.18	N/A
EF2_HUMAN	P13639	Eukaryotic translation elongation factor 2(EEF2)	3 N, 18 N, 84 N, 87 N, 101 N, 158 N, 184 N, 186 N, 202 N, 493 N, 673 N, 764 N	95,277	6.41	0.07	0.03
LEG3_HUMAN	P17931	Galectin 3(LGALS3)	4 N, 18 N, 119 N, 153 N, 164 N, 179 N	26,136	8.57	0.27	0.13
GANAB_HUMAN	Q14697	Glucosidase II alpha subunit(GANAB)	97 N, 265 N, 281 N, 317 N, 402 N, 420 N, 529 N	106,807	5.74	0.48	N/A
HS90A_HUMAN	P07900	Heat shock protein 90 alpha family class A member 1(HSP90AA1)	40 N, 51 N, 83 N, 106 N, 291 N, 354 N, 366 N, 373 N, 397 N, 415 N, 570 N	84,607	4.94	0.04	0.04
ENPL_HUMAN	P14625	Heat shock protein 90 beta family member 1(HSP90B1)	96 N, 129 N, 143 N, 162 N, 217 N, 228 N, 239 N, 431 N, 445 N, 491 N, 590 N, 748 N	92,411	4.76	1.08	0.57
GRP78_HUMAN	P38646	Heat shock protein family A (Hsp70) member 5(HSPA5)	177 N, 219 N, 239 N 282 N, 321 N, 5016 N, 520 N	72,288	5.07	0.86	0.31
HSP7C_HUMAN	P11142	Heat shock protein family A (Hsp70) member 8(HSPA8)	96 N, 194 N, 235 N, 239 N, 306 N, 364 N, 387 N, 454 N, 487 N	70,854	5.37	0.2	N/A
GRP75_HUMAN	P38646	Heat shock protein family A (Hsp70) member 9(HSPA9)	31 N, 64 N, 149 N, 180 N, 188 N, 343 N, 459 N	73,635	5.87	0.19	0.09
HSPB1_HUMAN	P04792	Heat shock protein family B (small) member 1(HSPB1)	#N/A	22,768	5.98	1.62	0.32
CH60_HUMAN	P10809	Heat shock protein family D (Hsp60) member 1(HSPD1)	177 N, 184 N, 208 N, 289 N, 308 N, 333 N, 426 N, 482 N	61,016	5.7	0.11	N/A
HNRPK_HUMAN	P61978	Heterogeneous nuclear ribonucleoprotein K(HNRNPK)	38 N, 134 N, 173 N, 453 N	50,944	5.39	0.21	N/A
NEST_HUMAN	P48681	Nestin(NES)	16 N, 112 N, 310 N, 530 N, 642 N, 653 N, 899 N, 902 N, 922 N, 981 N, 1001 N	177,332	4.35	N/A	0.02
PRDX1_HUMAN	Q06830	Peroxiredoxin 1(PRDX1)	14 N, 70 N, 101 N	22,096	8.27	0.76	0.15
PDIA1_HUMAN	P07237	Prolyl 4-hydroxylase subunit beta(P4HB)	33 N, 189 N, 215 N, 458 N	57,081	4.76	0.06	0.06
KPYM_HUMAN	P14618	Pyruvate kinase, muscle(PKM)	70 N, 163 N, 199 N, 210 N, 264 N, 318 N, 491 N, 498 N	57,900	7.96	0.06	0.18
RPN1_HUMAN	P04843	Ribophorin I(RPN1)	33 N, 97 N, 181 N, 190 N, 241 N, 247 N, 299 N, 335 N, 344 N, 380 N	68,527	5.96	0.15	0.05
TBB5_HUMAN	P07437	Tubulin beta class I(TUBB)		49,639	4.78	0.29	N/A

ConA lectin-binding cell lysates							
Cell Surface						EMPAI	
ID	Acession no.	Protein name	Net N glycosylated	Protein mass	PI	5-8F	6-10B
**B**							
ATPB_HUMAN	P06576	ATP synthase, H + transporting, mitochondrial F1 complex, beta polypeptide(ATP5B)	84 N, 146 N, 222 N, 244 N, 307 N	56,525	5.26	0.12	N/A
CD44_HUMAN	P16070	CD44 molecule (Indian blood group)(CD44)	25 N, 57 N, 110 N, 137 N, 509 N, 597 N, 599 N, 671 N	81,503	5.13	0.13	0.04
DAF_HUMAN	P08174	CD55 molecule (Cromer blood group)(CD55)	165 N, 303 N	41,374	7.79	0.08	N/A
L1CAM_HUMAN	P32004	L1 cell adhesion molecule(L1CAM)	118 N, 190 N, 192 N, 233 N, 247 N, 294 N, 316 N, 416 N, 479 N, 511 N, 671 N, 849 N, 945 N, 979 N	139,915	5.84	0.05	0.02
WNT3A_HUMAN	P56704	Wnt family member 3A(WNT3A)	58 N, 87 N, 175 N, 184 N	39,339	8.52	N/A	0.08
APMAP_HUMAN	Q9HDC9	Adipocyte plasma membrane associated protein(APMAP)	85 N, 126 N, 160 N, 176 N, 260 N, 326 N	46,451	5.82	0.07	0.07
PPB1_HUMAN	P05187	Alkaline phosphatase, placental(ALPP)	57 N, 106 N, 149 N, 187 N, 189 N, 219 N, 324 N, 417 N	57,917	5.87	1.29	0.64
ANXA1_HUMAN	P04083	Annexin A1(ANXA1)	146 N, 198 N, 219 N, 249 N	38,690	6.57	0.18	N/A
ANXA2_HUMAN	P07355	Annexin A2(ANXA2)	32 N, 41 N, 62 N, 258 N	38,580	7.57	N/A	0.09
FOLR1_HUMAN	P15328	Folate receptor 1(FOLR1)	35 N, 39 N, 87 N, 222 N	29,799	8.3	0.89	N/A
HS90A_HUMAN	P07900	Heat shock protein 90 alpha family class A member 1(HSP90AA1)	40 N, 51 N, 83 N, 106 N, 291 N, 354 N, 366 N, 373 N, 397 N, 415 N, 570 N	84,607	4.94	0.04	0.04
HS90B_HUMAN	P08238	Heat shock protein 90 alpha family class B member 1(HSP90AB1)	35 N, 78 N, 101 N, 283 N, 346 N, 352 N, 389 N, 562 N	83,212	4.97	0.12	0.08
HSP72_HUMAN	P54652	Heat shock protein family A (Hsp70) member 2(HSPA2)	197 N, 283 N, 259 N, 309 N, 390 N, 457 N, 490 N	69,978	5.56	0.15	N/A
GRP78_HUMAN	P38646	Heat shock protein family A (Hsp70) member 5(HSPA5)	177 N, 219 N, 239 N 282 N, 321 N, 5016 N, 520 N	72,288	5.07	0.86	0.31
CH60_HUMAN	P10809	Heat shock protein family D (Hsp60) member 1(HSPD1)	177 N, 184 N, 208 N, 289 N, 308 N, 333 N, 426 N, 482 N	61,016	5.7	0.11	N/A
ITB1_HUMAN	P05556	Integrin subunit beta 1(ITGB1)	25 N, 31 N, 92 N, 161 N, 205 N, 212 N, 227 N, 244 N, 269 N, 386 N, 417 N, 543 N, 557 N, 669 N, 708 N	88,357	5.27	0.04	N/A
1A34_HUMAN	P30453	Major histocompatibility complex, class I, A(HLA-A)	87 N, 110 N, 151 N	41,029	5.89	0.47	N/A
1B59_HUMAN	Q29940	Major histocompatibility complex, class I, B(HLA-B)	101 N, 110 N, 151 N	40,559	5.9	0.17	N/A
1C04_HUMAN	P30504	Major histocompatibility complex, class I, C(HLA-C)	101 N, 110 N, 151 N	40,969	6.04	0.08	N/A
HLAE_HUMAN	P13747	Major histocompatibility complex, class I, E(HLA-E)	98 N, 107 N, 148 N, 169 N	40,132	5.6	0.08	N/A
PDIA3_HUMAN	P30101	Protein disulfide isomerase family A member 3(PDIA3)	34 N, 90 N, 177 N, 181 N, 188 N, 194 N, 199 N, 239 N, 259 N, 277 N, 360 N, 374 N, 463 N	56,747	5.98	0.06	0.06
4F2_HUMAN	P08195	Solute carrier family 3 member 2(SLC3A2)	240 N, 352 N, 424 N, 428 N	67,952	4.89	0.05	N/A
TFR1_HUMAN	P02786	Transferrin receptor(TFRC)	47 N, 50 N, 55 N, 148 N, 150 N, 164 N, 198 N, 215 N, 251 N, 331 N, 372 N 379 ,N, 388 N, 483 N, 493 N, 727 N	84,818	6.18	0.26	0.26

**Table 2:** Peptides in identified in WGA-enriched fraction from 5-8F and 6-10B cell lysates. (**A**) Extracellular matrix-associated lectin-binding proteins involved in metastasis and (**B**) Cell surface- associated lectin-binding proteins involved in metastasis.

WGA lectin-binding cell lysates							
Extracellular matrix						EMPAI	
ID	Acession No	Protein name	Net N glycosylated	Protein mass	PI	5-8F	6-10B
**A**							
ATPA_HUMAN	P25705	ATP synthase, H + transporting, mitochondrial F1 complex, alpha subunit 1, cardiac muscle(ATP5A1)	40 N, 89 N, 113 N, 384 N, 409 N,	59,714	9.16	N/A	0.06
ACTG_HUMAN	P63261	Actin gamma 1(ACTG1)	12 N, 78 N, 111 N, 115 N, 296 N	41,766	5.31	N/A	0.7
CATD_HUMAN	P07339	Cathepsin D(CTSD)	73 N, 102 N, 134 N, 217 N, 227 N, 263 N	44,524	6.1	N/A	0.07
GANAB_HUMAN	Q14697	Clucosidase II alpha subunit(GANAB)	97 N, 265 N, 281 N, 317 N, 402 N, 420 N, 529 N	106,807	5.74	0.13	0.06
ENPL_HUMAN	P14625	Heat shock protein 90 beta family member 1(HSP90B1)	96 N, 129 N, 143 N, 162 N, 217 N, 228 N, 239 N, 431 N, 445 N, 491 N, 590 N, 748 N	92,411	4.76	0.28	0.11
GRP78_HUMAN	P38646	Heat shock protein family A (Hsp70) member 5(HSPA5)	177 N, 219 N, 239 N 282 N, 321 N, 5016 N, 520 N	72,288	5.07	0.19	0.14
HSP7C_HUMAN	P11142	Heat shock protein family A (Hsp70) member 8(HSPA8)	96 N, 194 N, 235 N, 239 N, 306 N, 364 N, 387 N, 454 N, 487 N	70,854	5.37	N/A	0.05
GRP75_HUMAN	P38646	Heat shock protein family A (Hsp70) member 9(HSPA9)	31 N, 64 N, 149 N, 180 N, 188 N, 343 N, 459 N	73,635	5.87	N/A	0.09
HSPB1_HUMAN	P04792	Heat shock protein family B (small) member 1(HSPB1)	#N/A	22,768	5.98	0.32	0.32
NEST_HUMAN	P48681	Nestin(NES)	16 N, 112 N, 310 N, 530 N, 642 N, 653 N, 899 N, 902 N, 922 N, 981 N, 1001 N	177,332	4.35	N/A	0.02
KPYM_HUMAN	P14618	Pyruvate kinase, muscle(PKM)	70 N, 163 N, 199 N, 210 N, 264 N, 318 N, 491 N, 498 N	57,900	7.96	0.12	0.25

WGA lectin-binding cell lysates							
Cell Surface						EMPAI	
ID	Acession no.	Protein name	Net N glycosylated	Protein mass	PI	5-8F	6-10B
**B**							
CD44_HUMAN	P16070	CD44 molecule (Indian blood group)(CD44)	25 N, 57 N, 110 N, 137 N, 509 N, 597 N, 599 N, 671 N	81,503	5.13	0.13	0.08
DAF_HUMAN	P08174	CD55 molecule (Cromer blood group)(CD55)	165 N, 303 N	41,374	7.79	0.26	0.26
L1CAM_HUMAN	P32004	L1 cell adhesion molecule(L1CAM)	118 N, 190 N, 192 N, 233 N, 247 N, 294 N, 316 N, 416 N, 479 N, 511 N, 671 N, 849 N, 945 N, 979 N	139,915	5.84	0.05	0.07
PPB1_HUMAN	P05187	Alkaline phosphatase, placental(ALPP)	57 N, 106 N, 149 N, 187 N, 189 N, 219 N, 324 N, 417 N	57,917	5.87	N/A	0.56
ANXA1_HUMAN	P04083	Annexin A1(ANXA1)	146 N, 198 N, 219 N, 249 N	57,917	5.87	0.18	N/A
CSPG4_HUMAN	Q6UVK1	Chondroitin sulfate proteoglycan 4(CSPG4)	36 N, 176 N, 361 N, 772 N, 833 N, 904 N, 1645 N, 1710 N, 2221 N	250,342	5.27	N/A	0.21
CR1_HUMAN	P17927	Complement C3b/C4b receptor 1 (Knops blood group)(CR1)	56 N, 112 N, 156 N, 181 N, 259 N, 284 N, 397 N, 378 N, 429 N, 447 N, 502 N, 631 N, 709 N, 734 N, 817 N, 825 N, 879 N, 897 N, 1071 N, 1184 N, 1275 N, 1311 N, 1728 N, 1856 N	223,517	6.57	N/A	0.01
FZD10_HUMAN	Q9ULW2	Frizzled class receptor 10(FZD10)	54 N, 60 N, 130 N, 132 N, 453 N, 468 N, 485 N	65,292	8.83	N/A	0.05
HS90B_HUMAN	P08238	Heat shock protein 90 alpha family class B member 1(HSP90AB1)	35 N, 78 N, 101 N, 283 N, 346 N, 352 N, 389 N, 562 N	83,212	4.97	0.04	N/A
GRP78_HUMAN	P38646	Heat shock protein family A (Hsp70) member 5(HSPA5)	177 N, 219 N, 239 N 282 N, 321 N, 5016 N, 520 N	72,288	5.07	0.19	0.14
ITA3_HUMAN	P26006	Integrin subunit alpha 3(ITGA3)	34 N, 86 N, 107 N, 167 N, 223 N, 247 N, 507 N, 511 N, 560 N, 619 N, 659 N, 951 N	118,680	6.51	N/A	0.03
ITA5_HUMAN	P08648	Integrin subunit alpha 5(ITGA5)	43 N, 182 N, 297 N, 307 N, 578 N, 609 N, 649 N, 675 N, 724 N	114,465	5.5	N/A	0.09
LDLR_HUMAN	P01130	Low density lipoprotein receptor(LDLR)	80 N, 97 N, 169 N, 272 N, 322 N, 370 N, 425 N, 439 N, 487 N, 515 N, 548 N, 645 N, 674 N, 780 N, 817 N	95,314	4.86	N/A	0.03
ROBO1_HUMAN	Q9Y6N7	Roundabout guidance receptor 1(ROBO1)	38 N, 88 N, 151 N, 270 N, 391 N, 417 N, 432 N, 463 N, 586 N, 613 N, 746 N, 790 N, 807 N, 820 N, 966 N, 1029 N, 1115 N, 1565 N	180,818	5.7	N/A	0.02
4F2_HUMAN	P08195	Solute carrier family 3 member 2(SLC3A2)	240 N, 352 N, 424 N, 428 N	67,952	4.89	0.05	0.15
TFR1_HUMAN	P02786	Transferrin receptor(TFRC)	47 N, 50 N, 55 N, 148 N, 150 N, 164 N, 198 N, 215 N, 251 N, 331 N, 372 N 379 ,N, 388 N, 483 N, 493 N, 727 N	84,818	6.18	0.12	0.3

**Table 3:** Peptides in identified in ConA-enriched fraction from 5-8F and 6-10B culture medium. (**A**) Extracellular matrix-associated lectin-binding proteins involved in metastasis and (**B**) Cell surface-associated lectin-binding proteins involved in metastasis.

ConA lectin-binding culture medium							
Extracellular matrix						EMPAI	
ID	Acession no.	Protein name	Net N glycosylated	Protein mass	PI	5-8F	6-10B
**A**							
S10A9_HUMAN	P06702	S100 calcium binding protein A9(S100A9)	11 N, 33 N, 47 N, 69 N	13,234	5.71	0.26	N/A
ACTG_HUMAN	P63261	Actin gamma 1(ACTG1)	12 N, 78 N, 111 N, 115 N, 296 N	41,766	5.31	N/A	0.08
FETUA_HUMAN	P02765	Alpha 2-HS glycoprotein(AHSG)	31 N, 51 N, 61 N, 156 N	39,300	5.43	0.27	0.27
DCD_HUMAN	P81605	Dermcidin(DCD)	44 N	11,277	6.08	0.3	N/A
EF2_HUMAN	P13639	Eukaryotic translation elongation factor 2(EEF2)	3 N, 18 N, 84 N, 87 N, 101 N, 158 N, 184 N, 186 N, 202 N, 493 N, 673 N, 764 N	95,277	6.41	0.03	N/A
IF4A1_HUMAN	P60842	Eukaryotic translation initiation factor 4A1(EIF4A1)	12 N, 28 N, 139 N, 206 N, 252 N	46,125	5.32	0.07	N/A
FGFR2_HUMAN	P21802	Fibroblast growth factor receptor 2(FGFR2)	83 N, 123 N, 158 N, 228 N, 346 N, 441 N, 631 N, 662 N, 727 N	91,966	5.61	N/A	0.04
FBLN1_HUMAN	P23142	Fibulin 1(FBLN1)	98 N, 535 N	77,162	5.07	0.04	N/A
GANAB_HUMAN	Q14697	Glucosidase II alpha subunit(GANAB)	97 N, 265 N, 281 N, 317 N, 402 N, 420 N, 529 N	106,807	5.74	0.16	N/A
HS90A_HUMAN	P07900	Heat shock protein 90 alpha family class A member 1(HSP90AA1)	40 N, 51 N, 83 N, 106 N, 291 N, 354 N, 366 N, 373 N, 397 N, 415 N, 570 N	84,607	4.94	0.04	N/A
ENPL_HUMAN	P14625	Heat shock protein 90 beta family member 1(HSP90B1)	96 N, 129 N, 143 N, 162 N, 217 N, 228 N, 239 N, 431 N, 445 N, 491 N, 590 N, 748 N	92,411	4.76	0.42	N/A
GRP78_HUMAN	P38646	Heat shock protein family A (Hsp70) member 5(HSPA5)	177 N, 219 N, 239 N 282 N, 321 N, 5016 N, 520 N	72,288	5.07	0.49	N/A
HSP7C_HUMAN	P11142	Heat shock protein family A (Hsp70) member 8(HSPA8)	96 N, 194 N, 235 N, 239 N, 306 N, 364 N, 387 N, 454 N, 487 N	70,854	5.37	0.09	N/A
GRP75_HUMAN	P38646	Heat shock protein family A (Hsp70) member 9(HSPA9)	31 N, 64 N, 149 N, 180 N, 188 N, 343 N, 459 N	73,635	5.87	0.04	N/A
HSPB1_HUMAN	P04792	Heat shock protein family B (small) member 1(HSPB1)	#N/A	22,768	5.98	0.51	N/A
IBP7_HUMAN	Q16270	Insulin like growth factor binding protein 7(IGFBP7)	#N/A	29,111	8.25	0.11	N/A
PRDX1_HUMAN	Q16270	Peroxiredoxin 1(PRDX1)	14 N, 70 N, 101 N	22,096	8.27	0.15	N/A
KPYM_HUMAN	P14618	Pyruvate kinase, muscle(PKM)	70 N, 163 N, 199 N, 210 N, 264 N, 318 N, 491 N, 498 N	57,900	7.96	0.12	N/A
RPN1_HUMAN	P04843	Ribophorin I(RPN1)	33 N, 97 N, 181 N, 190 N, 241 N, 247 N, 299 N, 335 N, 344 N, 380 N	68,527	5.96	0.05	N/A
TBB5_HUMAN	P07437	Tubulin beta class I(TUBB)	43 N, 63 N, 139 N, 212 N, 239 N, 259 N	49,639	4.78	0.29	N/A

ConA lectin-binding culture medium							
Extracellular exosome						EMPAI	
ID	Acession no.	Protein name	Net N glycosylated	Protein mass	PI	5-8F	6-10B
**B**							
ADAM9_HUMAN	Q13443	ADAM metallopeptidase domain 9(ADAM9)	98 N, 109 N, 125 N, 179 N, 244 N, 254 N, 267 N, 269 N, 283 N, 331 N, 352 N, 396 N, 400 N, 404 N, 419 N, 487 N, 514 N, 544 N, 577 N, 631 N, 771 N	90,497	7.71	0.04	N/A
AT1A1_HUMAN	P05023	ATPase Na + /K + transporting subunit alpha 1(ATP1A1)	129 N, 174 N, 209 N, 235 N, 360 N, 384 N, 429 N, 497 N, 582 N, 649 N, 784 N, 771 N, 783 N, 951 N	112,824	5.33	0.09	N/A
CD44_HUMAN	P16070	CD44 molecule (Indian blood group)(CD44)	25 N, 57 N, 110 N, 137 N, 509 N, 597 N, 599 N, 671 N	81,503	5.13	0.13	N/A
DAF_HUMAN	P08174	CD55 molecule (Cromer blood group)(CD55)	165 N, 303 N	41,374	7.79	0.26	N/A
SPHM_HUMAN	P51688	N-sulfoglucosamine sulfohydrolase(SGSH)	24 N, 41 N, 62 N, 86 N, 142 N, 151 N, 199 N, 264 N, 284 N	56,659	6.46	0.06	N/A
OCRL_HUMAN	Q01968	OCRL, inositol polyphosphate-5-phosphatase(OCRL)	154 N, 159 N, 188 N, 196 N, 236 N, 248 N, 280 N, 354 N, 410 N, 424 N, 454 N, 507 N, 509 N, 627 N	104,138	6.13	0.03	N/A
POTEE_HUMAN	Q6S8J3	POTE ankyrin domain family member E(POTEE)	79 N, 183 N, 200 N, 204 N, 233 N, 249 N, 299 N, 328 N, 374 N, 408 N, 426 N, 432 N, , 555 N, 648 N, 689 N	121,286	5.83	0.14	N/A
POTEF_HUMAN	A5A3E0	POTE ankyrin domain family member F(POTEF)	79 N, 183 N, 200 N, 204 N, 233 N, 249 N, 299 N, 328 N, 374 N, 426 N, 432 N, 492 N, 555 N, 689 N	121,367	5.83	0.14	N/A
ACTBM_HUMAN	Q9BYX7	POTE ankyrin domain family member K, pseudogene(POTEKP)	12 N, 78 N, 115 N, 296 N	41,989	5.91	0.16	N/A
S10A8_HUMAN	P05109	S100 calcium binding protein A8(S100A8)	10 N, 25 N, 67 N	10,828	6.51	0.32	N/A
S10A9_HUMAN	P06702	S100 calcium binding protein A9(S100A9)	11 N, 33 N, 47 N, 69 N	13,234	5.71	0.26	N/A
NHRF1_HUMAN	O14745	SLC9A3 regulator 1(SLC9A3R1)	22 N, 63 N, 66 N, 133 B, 135 B, 203	38,845	5.55	0.18	N/A
TRAP1_HUMAN	Q12931	TNF receptor associated protein 1(TRAP1)	171 N, 279 N, 288 N, 338 N, 399 N, 496 N, 588 N, 694 N	80,060	8.3	0.04	N/A
UGDH_HUMAN	O60701	UDP-glucose 6-dehydrogenase(UGDH)	74 N, 121 N, 151 N, 212 N, 263 N, 292 N, 450 N	54,989	6.73	0.06	N/A
ACACA_HUMAN	Q13085	Acetyl-CoA carboxylase alpha(ACACA)	14 N, 35 N, 126 N, 183 N, 186 N, 189 N, 225 N, 280 N, 325 N, 534 N, 536 N, 606 N, 668 N, 749 N, 859 N, 913 N, 1017 N, 1098 N, 1174 N, 1190 N, 1224 N, 1341 N, 1384 N, 1796 N, 2166 N	265,385	5.95	0.01	N/A
ACTB_HUMAN	P60709	Actin beta(ACTB)	12 N, 78 N, 115 N, 296 N	41,710	5.29	0.7	N/A
ACTG_HUMAN	P63261	Actin gamma 1(ACTG1)	12 N, 78 N, 111 N, 115 N, 296 N	41,766	5.31	N/A	0.08
ACTS_HUMAN	P68133	Actin, alpha 1, skeletal muscle(ACTA1)	14 N, 80 N, 117 N, 130 N, 164 N	42,024	5.23	0.57	N/A
ACTA_HUMAN	P62736	Actin, alpha 2, smooth muscle, aorta(ACTA2)	14 N, 80 N, 117 N, 130 N, 164 N	41,982	5.23	0.58	0.08
ACTBL_HUMAN	Q562R1	Actin, beta like 2(ACTBL2)	4 N, 13 N, 116 N, 129 N, 200 N, 297	41,976	5.39	0.26	N/A
ACTH_HUMAN	P63267	Actin, gamma 2, smooth muscle, enteric(ACTG2)	13 N, 79 N, 116 N, 129 N, 163 N	41,850	5.31	0.58	N/A
CD166_HUMAN	Q13740	Activated leukocyte cell adhesion molecule(ALCAM)	91 N, 95 N, 120 N, 107 N, 163 N, 370 N, 389 N, 480 N	65,061	5.92	0.05	N/A
ALBU_HUMAN	P02768	Albumin(ALB)	42 N, 68 N, 85 N, 135 N, 154 N, 291 N, 410 N, 415 N, 429 N	69,321	5.92	0.15	0.1
AL9A1_HUMAN	P49189	Aldehyde dehydrogenase 9 family member A1(ALDH9A1)	52 N, 157 N, 175 N, 207 N, 290 N, 469 N	53,767	5.69	N/A	0.06
FETUA_HUMAN	P02765	Alpha 2-HS glycoprotein(AHSG)	31 N, 51 N, 61 N, 156 N	39,300	5.43	0.27	0.27
A2MG_HUMAN	P01023	Alpha-2-macroglobulin(A2M)	4 N, 55 N, 70 N, 159 N, 247 N, 381 N, 386 N, 424 N, 635 N, 644 N, 647 N, 851 N, 867 N, 869 N, 938 N, 976 N, 983 N, 991 N, 1009 N, 1233 N, 1377 N	163,189	6	0.04	0.04
ANXA1_HUMAN	P04083	Annexin A1(ANXA1)	146 N, 198 N, 219 N, 249 N	38,690	6.57	0.39	N/A
BST2_HUMAN	Q10589	Bone marrow stromal cell antigen 2(BST2)	46 N, 65 N, 141 N	19,756	5.43	0.17	N/A
CATZ_HUMAN	Q9UBR2	Cathepsin Z(CTSZ)	70 N, 74 N, 122 N, 174 N, 184 N, 209 N	33,846	6.7	0.32	0.1
TCPH_HUMAN	Q99832	Chaperonin containing TCP1 subunit 7(CCT7)	134 N, 241 N, 487 N	59,329	7.55	0.06	N/A
CSPG4_HUMAN	Q6UVK1	Chondroitin sulfate proteoglycan 4(CSPG4)	36 N, 176 N, 361 N, 772 N, 833 N, 904 N, 1645 N, 1710 N, 2221 N	250,342	5.27	0.09	N/A
CROCC_HUMAN	Q5TZA2	Ciliary rootlet coiled-coil, rootletin(CROCC)	112 N, 280 N, 413 N, 420 N, 481 N, 745 N, 1536 N	228,388	5.45	0.01	N/A
C1R_HUMAN	P00736	Complement C1r(C1R)	110 N, 167 N, 221 N, 348 N, 396 N, 474 N, 524 N	80,067	5.82	N/A	0.04
DCD_HUMAN	P81605	Dermcidin(DCD)	44 N	11,277	6.08	0.3	N/A
EPDR1_HUMAN	Q9UM22	Ependymin related 1(EPDR1)	73 N	25,421	6.32	0.13	N/A
EF1A1_HUMAN	P68104	Eukaryotic translation elongation factor 1 alpha 1(EEF1A1)	197 N, 284 N, 307 N, 311 N, 324 N	50,109	9.1	0.07	N/A
EF2_HUMAN	P13639	Eukaryotic translation elongation factor 2(EEF2)	3 N, 18 N, 84 N, 87 N, 101 N, 158 N, 184 N, 186 N, 202 N, 493 N, 673 N, 764 N	95,277	6.41	0.03	N/A
IF4A1_HUMAN	P60842	Eukaryotic translation initiation factor 4A1(EIF4A1)	12 N, 28 N, 139 N, 206 N, 252 N	46,125	5.32	0.07	N/A
FAS_HUMAN	P49327	Fatty acid synthase(FASN)	195 N, 1476 N	273,254	6.01	0.01	N/A
FBLN1_HUMAN	P23142	Fibulin 1(FBLN1)	98 N, 535 N	77,162	5.07	0.04	N/A
FBLN7_HUMAN	Q53RD9	Fibulin 7(FBLN7)	26 N, 67 N, 85 N, 124 N, 169 N, 246 N, 307 N	47,345	7.88	0.07	N/A
GANAB_HUMAN	Q14697	Glucosidase II alpha subunit(GANAB)	97 N, 265 N, 281 N, 317 N, 402 N, 420 N, 529 N	106,807	5.74	0.16	N/A
GSLG1_HUMAN	Q92896	Golgi glycoprotein 1(GLG1)	45 N, 134 N, 149 N, 161 N, 165 N, 210 N, 386 N, 479 N, 581 N, 724 N, 769 N, 907 N, 1037 N, 113 N	134,464	6.52	0.02	0.02
HS90A_HUMAN	P07900	Heat shock protein 90 alpha family class A member 1(HSP90AA1)	40 N, 51 N, 83 N, 106 N, 291 N, 354 N, 366 N, 373 N, 397 N, 415 N, 570 N	84,607	4.94	0.04	N/A
HS90B_HUMAN	P08238	Heat shock protein 90 alpha family class B member 1(HSP90AB1)	35 N, 78 N, 101 N, 283 N, 346 N, 352 N, 389 N, 562 N	83,212	4.97	0.08	N/A
ENPL_HUMAN	P14625	Heat shock protein 90 beta family member 1(HSP90B1)	96 N, 129 N, 143 N, 162 N, 217 N, 228 N, 239 N, 431 N, 445 N, 491 N, 590 N, 748 N	92,411	4.76	0.42	N/A
HSP72_HUMAN	P54652	Heat shock protein family A (Hsp70) member 2(HSPA2)	197 N, 283 N, 259 N, 309 N, 390 N, 457 N, 490 N	69,978	5.56	0.1	N/A
GRP78_HUMAN	P11021	Heat shock protein family A (Hsp70) member 5(HSPA5)	177 N, 219 N, 239 N 282 N, 321 N, 5016 N, 520 N	72,288	5.07	0.49	N/A
HSP76_HUMAN	P17066	Heat shock protein family A (Hsp70) member 6(HSPA6)	33 N, 196 N, 237 N, 456 N	70,984	5.81	0.09	N/A
HSP7C_HUMAN	P11142	Heat shock protein family A (Hsp70) member 8(HSPA8)	96 N, 194 N, 235 N, 239 N, 306 N, 364 N, 387 N, 454 N, 487 N	70,854	5.37	0.09	N/A
GRP75_HUMAN	P38646	Heat shock protein family A (Hsp70) member 9(HSPA9)	31 N, 64 N, 149 N, 180 N, 188 N, 343 N, 459 N	73,635	5.87	0.04	N/A
HSPB1_HUMAN	P04792	Heat shock protein family B (small) member 1(HSPB1)	#N/A	22,768	5.98	0.51	N/A
ROA1_HUMAN	P09651	Heterogeneous nuclear ribonucleoprotein A1(HNRNPA1)	50 N, 171 N, 215 N, 276 N, 317 N, 319 N	38,723	9.17	0.09	N/A
ROA2_HUMAN	P22626	Heterogeneous nuclear ribonucleoprotein A2/B1(HNRNPA2B1)	178 N, 255 N, 278 N, 294 N, 299 N, 305 N	37,407	8.97	0.09	N/A
HORN_HUMAN	Q86YZ3	Hornerin(HRNR)	29 N, 39 N, 49 N, 66 N, 120 N, 170 N, 183 N, 204 N	282,228	10.05	0.01	0.01
HYOU1_HUMAN	Q9Y4L1	Hypoxia up-regulated 1(HYOU1)	61 N, 77 N, 277 N, 288 N, 314 N, 465 N, 515 N, 579 N, 657 N, 907 N	111,266	5.16	0.03	N/A
IBP7_HUMAN	Q16270	Insulin like growth factor binding protein 7(IGFBP7)	#N/A	29,111	8.25	0.11	N/A
ITAV_HUMAN	P06756	Integrin subunit alpha V(ITGAV)	74 N, 296 N, 704 N	115,964	5.45	0.03	N/A
ITB1_HUMAN	P05556	Integrin subunit beta 1(ITGB1)	25 N, 31 N, 92 N, 161 N, 205 N, 212 N, 227 N, 244 N, 269 N, 386 N, 417 N, 543 N, 557 N, 669 N, 708 N	88,357	5.27	0.08	N/A
ITIH2_HUMAN	P19823	Inter-alpha-trypsin inhibitor heavy chain 2(ITIH2)	24 N, 49 N, 103 N, 236 N, 387 N, 404 N, 441 N, 445 N, 491 N, 500 N, 590 , 719 N, 729 N, 741 N,	106,397	6.4	0.03	0.03
ITCH_HUMAN	Q96J02	Itchy E3 ubiquitin protein ligase(ITCH)	32 N, 58 N, 59 N, 61 N, 109 N, 110 N, 219 N, 396 N, 422 N, 451 N, 460 N, 518 N, 619 N, 723 N, 726 N	102,738	5.94	0.03	N/A
TRFL_HUMAN	P02788	Lactotransferrin(LTF)	44 N, 77 N, 145 N, 187 N, 280 N, 349 N, 433 N, 534 N, 557 N, 692 N	78,132	8.5	0.04	0.04
LRRK2_HUMAN	Q5S007	Leucine rich repeat kinase 2(LRRK2)	59 N, 130 N, 237 N, 246 N, 251 N, 281 N, 326 N, 335 N, 375 N, 376 N, 429 N, 431 N, 444 N, 531 N, 551 N, 693 N, 763 N, 834 N, 1093 N, 1101 N, 1135 N, 1221 N, 1255 N, 13,333 N, 1391 N, 1475 N, 1489 N, 1687 N, 1842 N, 2149 N	285,874	6.34	0.01	N/A
LAMP1_HUMAN	P11279	Lysosomal associated membrane protein 1(LAMP1)	35 N, 37 N, 76 N, 84 N, 103 N, 121 N, 165 N, 241 N, 249 N, 307 N, 322 N, 380 N	44,854	9	0.07	N/A
LAMP2_HUMAN	P13473	Lysosomal associated membrane protein 2(LAMP2)	32 N, 58 N, 75 N, 84 N, 104 N, 123 N, 148 N, 179 N, 227 N, 242 N, 300 N, 307 N	44,932	5.35	0.07	N/A
NEP_HUMAN	P08473	Membrane metalloendopeptidase(MME)	72 N, 202 N, 301 N, 325 N, 369 N, 416 N, 418 N, 491 N, 501 N, 514 N	85,460	5.54	0.04	N/A
MP2K1_HUMAN	Q02750	Mitogen-activated protein kinase kinase 1(MAP2K1)	21 N, 78 N, 195 N, 199 N, 399 N	43,411	6.18	N/A	0.08
MUC13_HUMAN	Q9H3R2	Mucin 13, cell surface associated(MUC13)	20 N, 59 N, 111 N, 113 N, 127 N, 142 N, 169 N, 284 N, 476 N	54,569	4.91	0.26	N/A
MEGF8_HUMAN	Q7Z7M0	Multiple EGF like domains 8(MEGF8)	50 N, 54 N, 127 N, 129 N, 217 N, 458 N, 1026 N, 1048 N, 1093 N, 1271 N, 1538 N, 1643 N, 2066 N, 2229 N, 2486 N	302,902	6.45	0.01	0.01
NUMA1_HUMAN	Q14980	Nuclear mitotic apparatus protein 1(NUMA1)	16 N, 142 N, 144 N, 240 N, 265 N, 314 N, 320 N, 428 N, 429 N, 511 N, 1036 N, 1628 N, 2016 N	238,115	5.63	0.01	N/A
PRDX1_HUMAN	Q06830	Peroxiredoxin 1(PRDX1)	14 N, 70 N, 101 N	22,096	8.27	0.15	N/A
PRDX2_HUMAN	P32119	Peroxiredoxin 2(PRDX2)	100 N, 186 N	21,878	5.66	0.15	N/A
PEX1_HUMAN	O43933	Peroxisomal biogenesis factor 1(PEX1)	44 N, 72 N, 88 N, 187 N, 285 N, 351 N, 375 N, 396 N, 423 N, 452 N, 521 N, 560 N, 592 N, 639 N, 751 N, 830 N, 863 N, 868 N, 901 N, 953 N, 1018 N, 1053 N	142,778	5.91	0.02	0.02
SERA_HUMAN	O43175	Phosphoglycerate dehydrogenase(PHGDH)	5 N, 35 N82 N, 102 N, 218 N, 220 N, 368 N, 449 N	56,614	6.29	0.06	N/A
PLS4_HUMAN	Q13085	Phospholipid scramblase 4(PLSCR4)	18 N, 34 N, 121 N, 141 N, 424 N	36,981	5.53	0.09	N/A
PCBP1_HUMAN	Q15365	Poly(rC) binding protein 1(PCBP1)	11 N, 53 N, 289 N	37,474	6.66	0.09	N/A
PCBP2_HUMAN	Q15366	Poly(rC) binding protein 2(PCBP2)	11 N, 53 N, 66 N, 89 N	38,556	6.33	0.09	N/A
PCBP3_HUMAN	P57721	Poly(rC) binding protein 3(PCBP3)	43 N, 279 N	35,916	8.22	0.09	N/A
PIP_HUMAN	P12273	Prolactin induced protein(PIP)	26 N, 31 N, 49 N, 83 N, 105 N	16,562	8.26	0.2	N/A
PDIA3_HUMAN	P30101	Protein disulfide isomerase family A member 3(PDIA3)	34 N, 90 N, 177 N, 181 N, 188 N, 194 N, 199 N, 239 N, 259 N, 277 N, 360 N, 374 N, 463 N	56,747	5.98	0.06	N/A
PDIA6_HUMAN	Q15084	Protein disulfide isomerase family A member 6(PDIA6)	18 N, 36 N, 109 N	48,091	4.95	0.07	N/A
PTN13_HUMAN	Q12923	Protein tyrosine phosphatase, non-receptor type 13(PTPN13)	179 N, 234 N, 253 N, 396 N, 472 N, 570 N, 580 N, 621 N, 653 N, 752 N, 892 N, 918 N, 984 N, 1148 N, 1376 N, 1422 N, 1685 N, 1852 N, 1935 N, 2034 N, 2091 N	276,733	5.99	0.01	N/A
PTPRD_HUMAN	P23468	Protein tyrosine phosphatase, receptor type D(PTPRD)	172 N, 211 N, 22 N, 254 N, 287 N, 4455 N, 470 N, 521 N, 639 N, 707 N, 899 N, 824 N, 1043 N, 1136 N, 1216 N, 1380 N, 1388 N, 1669 N	214,625	6.14	0.02	N/A
PTPRF_HUMAN	P10586	Protein tyrosine phosphatase, receptor type F(PTPRF)	181 N, 211 N, 222 N, 250 N, 393 N, 441 N, 721 N, 921 N, 966 N, 1049 N, 1211 N, 1375 N, 1383 N, 1664 N	212,744	5.93	0.02	N/A
KPYM_HUMAN	P14618	Pyruvate kinase, muscle(PKM)	70 N, 163 N, 199 N, 210 N, 264 N, 318 N, 491 N, 498 N	57,900	7.96	0.12	N/A
SERPH_HUMAN	P50454	Serpin family H member 1(SERPINH1)	66 N, 120 N, 202 N, 244 N	46,411	8.75	0.07	N/A
GTR1_HUMAN	P11166	Solute carrier family 2 member 1(SLC2A1)	29 N, 45 N, 88 N, 94 N, 100 N, 182 N, 217 N, 219 N	54,049	8.93	0.06	N/A
4F2_HUMAN	P08195	Solute carrier family 3 member 2(SLC3A2)	240 N, 352 N, 424 N, 428 N	67,952	4.89	0.05	N/A
CTL1_HUMAN	Q8WWI5	Solute carrier family 44 member 1(SLC44A1)	67 N, 99 N, 494 N, 560 N	73,253	8.93	N/A	0.04
SPTN5_HUMAN	Q9NRC6	Spectrin beta, non-erythrocytic 5(SPTBN5)	79 N, 138 N, 196 N, 198 N, 237 N, 647 N, 1235 N, 1347 N, 1553 N, 2303 N	416,579	6.23	0.02	N/A
ST1C2_HUMAN	O00338	Sulfotransferase family 1C member 2(SULT1C2)	65 N, 165 N	34,857	7.12	N/A	0.1
TFR1_HUMAN	P02786	Transferrin receptor(TFRC)	47 N, 50 N, 55 N, 148 N, 150 N, 164 N, 198 N, 215 N, 251 N, 331 N, 372 N 379 ,N, 388 N, 483 N, 493 N, 727 N	84,818	6.18	0.26	0.04
TPP1_HUMAN	O14773	Tripeptidyl peptidase 1(TPP1)	57 N, 84 N, 210 N, 401 N	61,210	6.01	0.11	N/A
TPM3_HUMAN	P06753	Tropomyosin 3(TPM3)	90 N, 200 N	32,799	4.68	0.21	N/A
TPM4_HUMAN	P67936	Tropomyosin 4(TPM4)	55 N, 163 N, 231 N	28,504	4.67	0.12	N/A
TBB1_HUMAN	Q9H4B7	Tubulin beta 1 class VI(TUBB1)	14 N, 115 N, 153 N, 165 N, 226 , 247 N, 348 N	50,295	5.05	0.07	N/A
TBB2A_HUMAN	Q13885	Tubulin beta 2A class IIa(TUBB2A)	18 N, 248 N, 349 N, 374 N, 445 N, 486 N	49,875	4.78	0.14	N/A
TBB3_HUMAN	Q13509	Tubulin beta 3 class III(TUBB3)	14 N, 126 N, 165 N, 195 N, 226 N, 348 N, 370 N	50,400	4.83	0.07	N/A
TBB5_HUMAN	P07437	Tubulin beta class I(TUBB)	43 N, 63 N, 139 N, 212 N, 239 N, 259 N	49,639	4.78	0.29	N/A
VASN_HUMAN	Q6EMK4	Vasorin(VASN)	62 N, 104 N, 112 N, 158 N, 179 N, 226 N	71,668	7.16	0.05	N/A

**Table 4:** Peptides in identified in WGA-enriched fraction from 5-8F and 6-10B culture medium. (**A**) Extracellular matrix-associated lectin-binding proteins involved in metastasis and (**B**) Cell surface-associated lectin-binding proteins involved in metastasis.

WGA lectin-binding culture medium							
Extracellular matrix						EMPAI	
ID	Acession no.	Protein name	Net N glycosylated	Protein mass	PI	5-8F	6-10B
**A**							
ATS10_HUMAN	Q9H324	ADAM metallopeptidase with thrombospondin type 1 motif 10(ADAMTS10)	90 N, 222 N, 285 N, 3334 N, 566 N, 998 N	120,796	8.34	0.03	N/A
POZP3_HUMAN	Q6PJE2	POM121 and ZP3 fusion(POMZP3)	73 N	23,181	6.1	0.14	N/A
ACTG_HUMAN	P63261	Actin gamma 1(ACTG1)	12 N, 78 N, 111 N, 115 N, 296 N	41,766	5.31	0.84	0.58
FETUA_HUMAN	P02765	Alpha 2-HS glycoprotein(AHSG)	31 N, 51 N, 61 N, 156 N	39,300	5.43	0.91	0.91
CLUS_HUMAN	P10909	Clusterin(CLU)	43 N, 51 N,, 86 N, 103 N, 155 N, 279 N, 291 N	52,461	5.89	N/A	0.13
DCD_HUMAN	P81605	Dermcidin(DCD)	44 N	11,277	6.08	N/A	0.3
FBN2_HUMAN	P35556	Fibrillin 2(FBN2)	87 N, 108 N, 117 N, 152 N, 156 N, 291 N, 372 N, 440 N, 447 N, 449 N, 465 N, 503 N, 546 N, 588 N, 625 N, 652 N, 670 N, 786 N, 823 N, 863 N, 869 N, 901 N, 957 N, 965 N, 1060 N, 1209 ,N 1213 N, 1331 N, 13,777 N, 1386 N, 1467 N, 1499 N, 1741 N, 1740 N, 1767 N, 1773 N, 1821 N, 1901 N, 1945 N, 2184 N, 2225 N, 2351 N, 2462 N	314,558	4.73	N/A	0.01
FBLN1_HUMAN	P23142	Fibulin 1(FBLN1)	98 N, 535 N	77,162	5.07	0.09	0.04
LG3BP_HUMAN	Q08380	Galectin 3 binding protein(LGALS3BP)	20 N, 33 N, 51 N, 69 N, 112 N, 162 N, 179 N, 192 N, 258 N, 362 N, 567 N	65,289	5.13	0.01	N/A
G3P_HUMAN	P04406	Glyceraldehyde-3-phosphate dehydrogenase(GAPDH)	9 N, 24 N, 41 N, 64 N, 167 N, 225 N, 322 N	36,030	8.57	0.55	0.3
HS90A_HUMAN	P07900	Heat shock protein 90 alpha family class A member 1(HSP90AA1)	40 N, 51 N, 83 N, 106 N, 291 N, 354 N, 366 N, 373 N, 397 N, 415 N, 570 N	84,607	4.94	N/A	0.08
PEDF_HUMAN	P36955	Serpin family F member 1(SERPINF1)	52 N, 77 N	46,313	5.97	0.07	0.15
VTNC_HUMAN	P04004	Vitronectin(VTN)	33 N, 86 N, 253 N, 416 N, 448 N	54,271	5.55	N/A	0.06

WGA lectin-binding culture medium							
Extracellular exosome						EMPAI	
ID	Acession no.	Protein name	Net N glycosylated	Protein mass	PI	5-8F	6-10B
**B**							
GBG7_HUMAN	O60262	G protein subunit gamma 7(GNG7)	#N/A	7517	8.71	N/A	0.47
POTEE_HUMAN	Q6S8J3	POTE ankyrin domain family member E(POTEE)	79 N, 183 N, 200 N, 204 N, 233 N, 249 N, 299 N, 328 N, 374 N, 408 N, 426 N, 432 N, , 555 N, 648 N, 689 N	121,286	5.83	0.14	0.11
POTEF_HUMAN	A5A3E0	POTE ankyrin domain family member F(POTEF)	79 N, 183 N, 200 N, 204 N, 233 N, 249 N, 299 N, 328 N, 374 N, 426 N, 432 N, 492 N, 555 N, 689 N	121,367	5.83	0.14	0.11
ACTBM_HUMAN	Q9BYX7	POTE ankyrin domain family member K, pseudogene(POTEKP)	12 N, 78 N, 115 N, 296 N	41,989	5.91	0.16	N/A
TRAP1_HUMAN	Q12931	TNF receptor associated protein 1(TRAP1)	171 N, 279 N, 288 N, 338 N, 399 N, 496 N, 588 N, 694 N	80,060	8.3	N/A	0.04
ACTG_HUMAN	P63261	Actin gamma 1(ACTG1)	12 N, 78 N, 111 N, 115 N, 296 N	41,766	5.31	0.84	0.58
ACTA_HUMAN	P62736	Actin, alpha 2, smooth muscle, aorta(ACTA2)	14 N, 80 N, 117 N, 130 N, 164 N	41,982	5.23	0.46	N/A
ACTBL_HUMAN	Q562R1	Actin, beta like 2(ACTBL2)	4 N, 13 N, 116 N, 129 N, 200 N, 297	41,976	5.39	0.26	0.16
ACTH_HUMAN	P63267	Actin, gamma 2, smooth muscle, enteric(ACTG2)	13 N, 79 N, 116 N, 129 N, 163 N	41,850	5.31	0.46	N/A
ALBU_HUMAN	P02768	Albumin(ALB)	42 N, 68 N, 85 N, 135 N, 154 N, 291 N, 410 N, 415 N, 429 N	69,321	5.92	0.26	0.32
FETUA_HUMAN	P02765	Alpha 2-HS glycoprotein(AHSG)	31 N, 51 N, 61 N, 156 N	39,300	5.43	0.91	0.91
A2ML1_HUMAN	A8K2U0	Alpha-2-macroglobulin like 1(A2ML1)	22 N, 32 N, 103 N, 120 N,M 164 N, 281 N, 332 N, 409 N, 685 N, 857 N, 974 N, 1020 N1034 N	161,001	5.48	0.02	N/A
A2MG_HUMAN	P01023	Alpha-2-macroglobulin(A2M)	4 N, 55 N, 70 N, 159 N, 247 N, 381 N, 386 N, 424 N, 635 N, 644 N, 647 N, 851 N, 867 N, 869 N, 938 N, 976 N, 983 N, 991 N, 1009 N, 1233 N, 1377 N	163,189	6	0.04	0.02
BASI_HUMAN	P35613	Basigin (Ok blood group)(BSG)	214 N, 289 N, 302 N	42,174	5.39	0.08	0.08
CLUS_HUMAN	P10909	Clusterin(CLU)	43 N, 51 N,, 86 N, 103 N, 155 N, 279 N, 291 N	52,461	5.89	N/A	0.13
C1R_HUMAN	P00736	Complement C1r(C1R)	110 N, 167 N, 221 N, 348 N, 396 N, 474 N, 524 N	80,067	5.82	N/A	0.04
DCD_HUMAN	P81605	Dermcidin(DCD)	44 N	11,277	6.08	N/A	0.3
FBLN1_HUMAN	P23142	Fibulin 1(FBLN1)	98 N, 535 N	77,162	5.07	0.09	0.04
FUCO2_HUMAN	Q9BTY2	Fucosidase, alpha-L- 2, plasma(FUCA2)	115 N, 151 N, 218 N, 239 N, 293 N, 363 N	54,032	5.84	N/A	0.06
LG3BP_HUMAN	Q08380	Galectin 3 binding protein(LGALS3BP)	20 N, 33 N, 51 N, 69 N, 112 N, 162 N, 179 N, 192 N, 258 N, 362 N, 567 N	65,289	5.13	0.01	N/A
GELS_HUMAN	P06396	Gelsolin(GSN)	50 N, 84 N, 105 N, 107 N, 118 N, 140 N, 233 N, 250 N, 254 N, 305 N, 416 N	85,644	5.9	N/A	0.04
G3P_HUMAN	P04406	Glyceraldehyde-3-phosphate dehydrogenase(GAPDH)	9 N, 24 N, 41 N, 64 N, 167 N, 225 N, 322 N	36,030	8.57	0.55	0.3
HS90A_HUMAN	P07900	Heat shock protein 90 alpha family class A member 1(HSP90AA1)	40 N, 51 N, 83 N, 106 N, 291 N, 354 N, 366 N, 373 N, 397 N, 415 N, 570 N	84,607	4.94	N/A	0.08
HS902_HUMAN	Q14568	Heat shock protein 90 alpha family class A member 2, pseudogene(HSP90AA2P)	40 N, 51 N, 83 N, 106 N, 290 N	39,340	4.57	N/A	0.08
HS90B_HUMAN	P08238	Heat shock protein 90 alpha family class B member 1(HSP90AB1)	35 N, 78 N, 101 N, 283 N, 346 N, 352 N, 389 N, 562 N	83,212	4.97	N/A	0.08
H90B2_HUMAN	Q58FF8	Heat shock protein 90 alpha family class B member 2, pseudogene(HSP90AB2P)	35 N, 101 N, 163 N, 205 N, 232 N, 335 N	44,321	4.79	N/A	0.07
ROA1_HUMAN	P09651	Heterogeneous nuclear ribonucleoprotein A1(HNRNPA1)	50 N, 171 N, 215 N, 276 N, 317 N, 319 N	38,723	9.17	N/A	0.09
H2A1A_HUMAN	Q96QV6	Histone cluster 1 H2A family member a(HIST1H2AA)	39 N, 69 N	14,225	10.86	N/A	0.24
HORN_HUMAN	Q86YZ3	Hornerin(HRNR)	29 N, 39 N, 49 N, 66 N, 120 N, 170 N, 183 N, 204 N	282,228	10.05	0.01	0.01
ITIH2_HUMAN	P19823	Inter-alpha-trypsin inhibitor heavy chain 2(ITIH2)	24 N, 49 N, 103 N, 236 N, 387 N, 404 N, 441 N, 445 N, 491 N, 500 N, 590 , 719 N, 729 N, 741 N,	106,397	6.4	0.06	0.03
LDH6A_HUMAN	Q6ZMR3	Lactate dehydrogenase A like 6A(LDHAL6A)	11 N, 21 N, 90 N, 123 N, 164 N, 229 N	36,484	6.51	0.09	N/A
LDHA_HUMAN	P00338	Lactate dehydrogenase A(LDHA)	11 N, 21 N, 84 N, 108 N, 113 N, 115 N, 123 N, 164 N, 205 N	36,665	8.44	0.54	0.3
LDHB_HUMAN	P07195	Lactate dehydrogenase B(LDHB)	109 N, 11 N, 116 N, 165 N, 206 N, 223 N	36,615	5.71	0.19	0.19
LDHC_HUMAN	P07864	Lactate dehydrogenase C(LDHC)	164 N, 205 N, 229 N, 309 N, 325 N	36,288	7.08	0.09	0.09
MEGF8_HUMAN	Q7Z7M0	Multiple EGF like domains 8(MEGF8)	50 N, 54 N, 127 N, 129 N, 217 N, 458 N, 1026 N, 1048 N, 1093 N, 1271 N, 1538 N, 1643 N, 2066 N, 2229 N, 2486 N	302,902	6.45	0.01	0.01
MYO5A_HUMAN	Q9Y4I1	Myosin VA(MYO5A)	88 N, 131 N, 191 N, 200 N, 216 N, 286 N, 239 N, 413 N, 418 N, 460 N, 533 N, 545 N, 664 N, 855 N, 927 N, 952 N, 958 N, 1080 N, 1319 N, 1412 N, 1722 N	215,285	8.7	0.02	N/A
NEBU_HUMAN	P20929	Nebulin(NEB)	#N/A	772,454	9.11	N/A	0.01
NUMA1_HUMAN	Q14980	Nuclear mitotic apparatus protein 1(NUMA1)	16 N, 142 N, 144 N, 240 N, 265 N, 314 N, 320 N, 428 N, 429 N, 511 N, 1036 N, 1628 N, 2016 N	238,115	5.63	N/A	0.01
PPIA_HUMAN	P62937	Peptidylprolyl isomerase A(PPIA)	71 N, 108 N, 137 N	18,001	7.68	N/A	0.19
PEX1_HUMAN	O43933	Peroxisomal biogenesis factor 1(PEX1)	44 N, 72 N, 88 N, 187 N, 285 N, 351 N, 375 N, 396 N, 423 N, 452 N, 521 N, 560 N, 592 N, 639 N, 751 N, 830 N, 863 N, 868 N, 901 N, 953 N, 1018 N, 1053 N	142,778	5.91	0.02	0.02
PGK1_HUMAN	P00558	Phosphoglycerate kinase 1(PGK1)	5 N, 26 N, 53 N, 121 N, 138 N, 180 N, 195 N	44,586	8.3	0.07	0.07
PGK2_HUMAN	P07205	Phosphoglycerate kinase 2(PGK2)	26 N, 53 N, 121 N	44,767	8.74	0.07	0.07
PSMD2_HUMAN	Q13200	Proteasome 26S subunit, non-ATPase 2(PSMD2)	199 N, 245 N, 329 N, 371 N, 378 N, 531 N, 605 N, 705 N	100,136	5.08	N/A	0.03
ANT3_HUMAN	P01008	Serpin family C member 1(SERPINC1)	4 N, 77 N, 105 N, 107 N, 128 N, 159 N, 167 N, 187 N	52,569	6.32	N/A	0.06
PEDF_HUMAN	P36955	Serpin family F member 1(SERPINF1)	52 N, 77 N	46,313	5.97	0.07	0.15
SHRM2_HUMAN	Q13796	Shroom family member 2(SHROOM2)	164 N, 234 N, 1551 N, 1565 N	176,303	6.64	0.02	N/A
4F2_HUMAN	P08195	Solute carrier family 3 member 2(SLC3A2)	240 N, 352 N, 424 N, 428 N	67,952	4.89	0.05	N/A
SPTN5_HUMAN	Q9NRC6	Spectrin beta, non-erythrocytic 5(SPTBN5)	79 N, 138 N, 196 N, 198 N, 237 N, 647 N, 1235 N, 1347 N, 1553 N, 2303 N	416,579	6.23	N/A	0.01
TFR1_HUMAN	P02786	Transferrin receptor(TFRC)	47 N, 50 N, 55 N, 148 N, 150 N, 164 N, 198 N, 215 N, 251 N, 331 N, 372 N 379 ,N, 388 N, 483 N, 493 N, 727 N	84,818	6.18	N/A	0.04
TBA1A_HUMAN	Q71U36	Tubulin alpha 1a(TUBA1A)	18 N, 248 N, 349 N, 374 N, 445 N, 486 N	50,104	4.94	0.07	N/A
VTNC_HUMAN	P04004	Vitronectin(VTN)	33 N, 86 N, 253 N, 416 N, 448 N	54,271	5.55	N/A	0.06

### Galectin-3 is associated with metastasis in NPC

**Figure 4:**
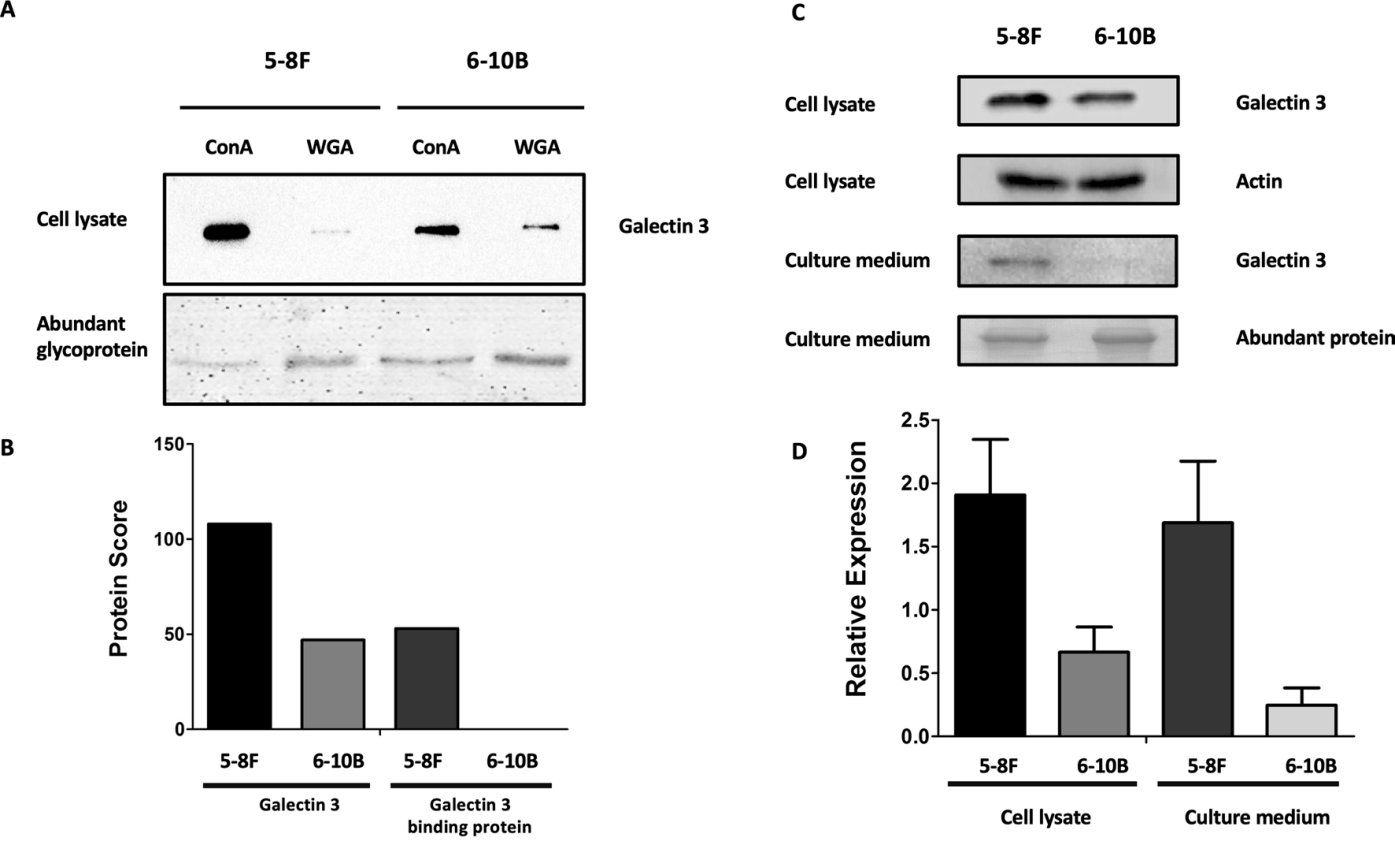
Differential expression of Galectin-3 in 5-8F and 6-10B NPC cells. (**A**) The expression of Galectin-3 in ConA-enriched and WGA-enriched fractions obtained from both cell types was validated using Western immunoblotting with Galectin-3 specific monoclonal antibody. (**B**) Protein score of Galectin-3 and Galectin-3 binding protein identified in each fraction. The level of Galectin-3 in 5-8F cells was higher than 6-10B cells in ConA-enriched fraction. Galectin-3 binding protein was only present in WGA-enriched fraction in 5-8F cells, but not 6-10B. (**C**) The expression of Galectin-3 was evaluated in cell lysates and culture medium from both cells types using Western immunoblotting with Galectin-3 specific monoclonal antibody. (**D**) A bar chart represents the quantitation of Galectin-3 expression in cell lysates and culture medium from 5-8F and 6-10B cells. Actin and abundant proteins were used to normalize as a relative of control.

To validate the differential expression of Galectin-3 in both types of NPC cells, Galectin-3 antibody was then probed to each fraction of proteins. The immunoblotting results were in accordance with the protein score obtained from the proteomic analysis (Fig. 4A,B). To further confirm these findings, we subsequently evaluated the expression of Galectin-3 in total whole cell lysates and culture medium from 5-8F and 6-10B cells. The results revealed that 5-8F cells and the medium derived thereof expressed Galectin-3 protein higher than 6-10B cells (Fig. 4C,D). Thus, the findings from both proteomic analyses and in vitro cell cultures were highly in agreement to each other.

**Figure 5:**
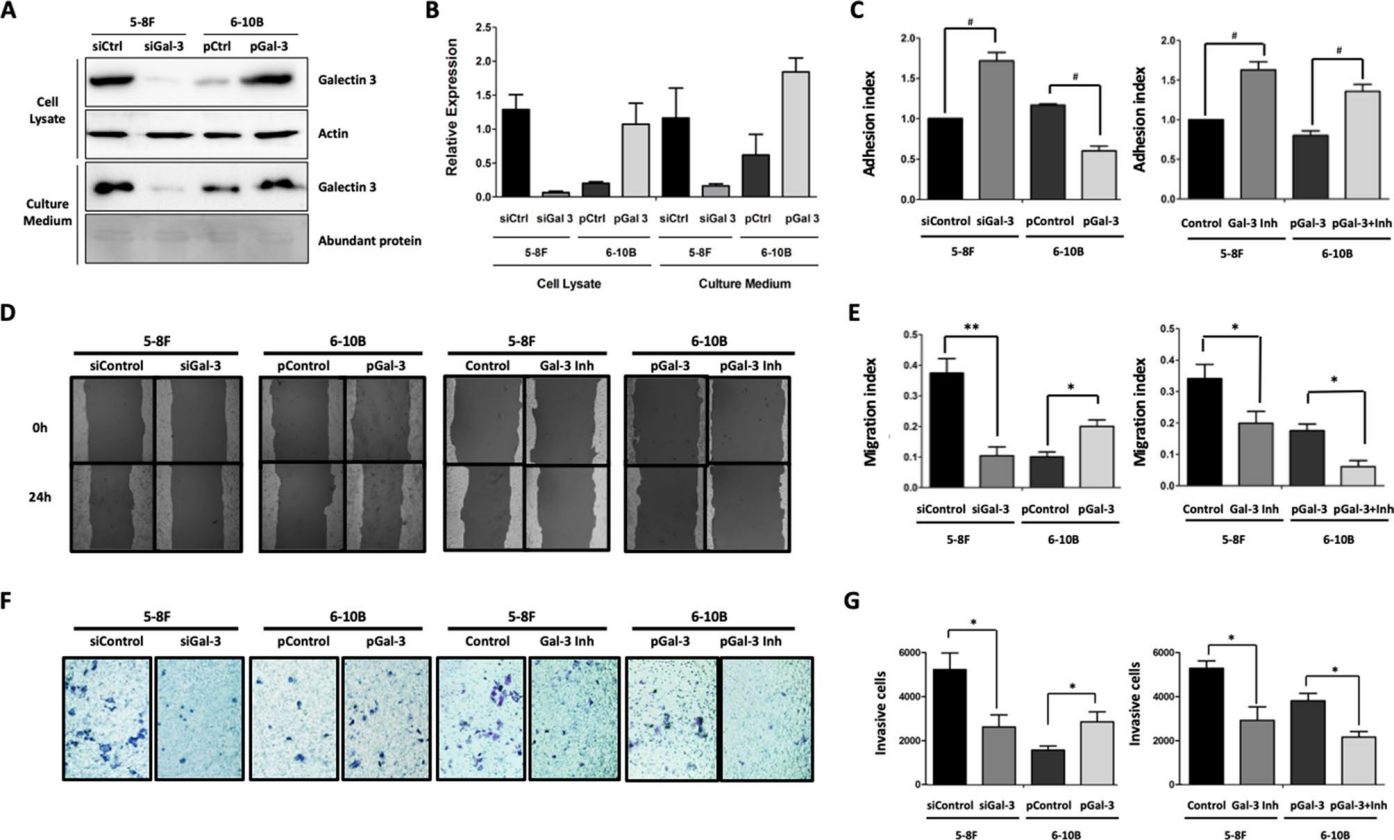
Galectin-3 contributes to metastatic phenotypes of NPC cells. Galectin-3 siRNA (siGal-3) and control siRNA (siControl) were transfected into 5-8F cells, while Galectin-3 expression (pGal3) and control (pControl) plasmids were transferred into 6-10B cells. Modified citrus pectin was used as a Galectin-3 inhibitor (Inh). (**A**) Immunoblotting detection of Galectin-3 in cell lysates and culture medium was performed to verify the galectin-3 knockdown in 5-8F and overexpression in 6-10B cells. (**B**) A bar chart represents the quantitation of Galectin-3 expression in cell lysates and culture medium from the Galectin-3 knockdown 5-8F and overexpressing 6-10B cells with controls. Actin and abundant proteins were used to normalize as a relative of control. (**C**) Adhesion index of cells after the knockdown or overexpression of Galectin-3 and treatment with MCP. (**D**) Representative photographs of cell migration by scratch wound assay. (**E**) Migration index of cells after the knockdown or overexpression of Galectin-3 and treatment with MCP. (**F**) Representative photographs of invasive cells by Matrigel invasion assay. (**G**) The number of invasive cells after the knockdown or overexpression of Galectin-3 and treatment with MCP. All data were from at least three experiments. Each bar represents the mean ± SEM *, *P* < 0.05; **, < 0.01 and # < 0.001.

To further investigate the functional role of Galectin-3 in NPC cells, the siRNA was introduced to the Galectin-3 highly expressed 5-8F cells and the *gal-3* expression plasmid was introduced to the Galectin-3 poorly expressed 6-10B cells. The immunoblotting analyses revealed that we successfully generated the *gal-3* knockdown 5-8F cells and the Galectin-3 overexpressing 6-10F cells (Fig. 5A,B). The phenotypic characterization on these cells, together with the treatment with modified citrus pectin as a Galectin-3 specific inhibitor was performed. The results demonstrated that the *gal-3* knockdown 5-8F cells exhibited higher ability to attach on a monolayer of an extracellular matrix compared to the control cells, while the Galectin-3 overexpressing 6-10B cells yielded the lower adhesive index compared to the 6-10B cells harboring the control plasmid. The treatment of Galectin-3 inhibitor promoted the cell adhesion in both 5-8F cells and Galectin-3 overexpressing 6-10B cells (Fig. 5C). Furthermore, Galectin-3 clearly enhanced migrative and invasive ability of NPC cells as the overexpression of Galectin-3 in 6-10B cells exalted its ability to migrate and invade, whereas the *gal-3* silencing in 5-8F cells and its inhibition in both 5-8F and Galectin-3 overexpressing 6-10B cells greatly reduced cell migration and invasion (Fig. 5D–G). Moreover, to elucidate the signaling pathways that might potentially be involved in Galectin-3 mediated metastatic phenotypes, the expression of certain signaling proteins were evaluated. We found down-regulation of active β-catenin, P38 and AKT proteins in the *gal-3* knockdown 5-8F cells with no changes for IKK and NF-κB. For Galectin-3 overexpressing 6-10B cells, up-regulation of active β-catenin was observed together with IKK and NF-κB (Fig. S1). Altogether, these results indicated that Galectin-3 modulates NPC cell metastatic phenotypes including adhesion, migration and invasion.

## Discussion

Tumor metastasis is one of the major causes of mortality in cancer patients. The metastatic process involves tumor cell extravasation and subsequent migration and invasion into adjacent tissues, all of which are tightly controlled by cell surface mechanisms^13^. It is evident that aberrations in certain glycosylated proteins play an important role in cancer metastasis^14^. Thus, the discovery of differentially expressed glycoproteins during the metastatic process will help to unravel the fundamental biomolecular activities associated with cancer metastasis and potentially can prompt us to explicate novel therapeutic targets. Recent advancements in MS technology have boosted high-throughput analyses of many proteins and glycoproteins. In this study, two immobilized lectin affinity chromatography (ConA and WGA) normally used to enrich glycoproteins were applied to two different biological systems. These lectins are commonly used for reproducibly enriching glycoproteins from serum or plasma^12^. ConA primarily recognizes alpha-linked mannose and terminal glucose residues, which are a part of the N-glycan core structure, while WGA exhibits a primary affinity to GlcNAc groups and bind to sialic acid^12^. The combination of separation techniques using lectin affinity chromatography and a suitable mass spectrometry in proteomic evaluation would simply provide higher discovery rate of glycoproteins as effectively and reproducibly used for proteomic studies^15^. As NPC occurs in a silent small area, patients are usually diagnosed at late stages with higher rates of metastasis. Hence, in this study, we performed the comparative proteomics of ConA- and WGA-enriched fractions in highly and lowly metastatic NPC cells in order to retrieve glycoprotein biomarkers responsible for NPC metastatic process. Glycoproteins in cell lysates and culture media were enriched by lectin affinity chromatography. After digestion and LC MS/MS analysis, the enrichment fraction profiling in both types of NPC cells were then elucidated.

In cancer cells, glycoproteins with mannose-rich glycan moieties may regulate key cellular processes, which drive oncogenes and corresponding proteins involved in potential cell progression, proliferation and cancer cell transformation^16^, while changes in sialylation may enhance integrin-mediated cellular adhesion or inhibit interactions to integrin- extracellular matrix, leading to potentially facilitating cancer aggressively spread and eventual metastasis^17^. Based on our data, many lectin-specific glycosylated proteins were identified only in highly metastatic 5-8F cells including UDP-glucose:glycoprotein glucosyltransferase 1 (UGGT1), dolichyl-diphosphooligosaccharide protein glycosyltransferase subunit 1 (RPN1), GDP-fucose protein O-fucosyltransferase 2 (POFUT2), and galactosylgalactosylxylosyl protein 3-beta-glucuronosyltransferase 3 (B3GAT1), whereas, only GDP-fucose protein O-fucosyltransferase 1 (POFUT1) was found in lowly metastatic 6-10B cells. It has been well established that glycosylation processing pathways are disturbed during carcinogenesis and tumor progression as resulted from aberrant activity of glycan modification enzymes, affecting various aspects of the biological behaviors of cancer cells including metastatic potential^18–20^. In accordance, differential expression of lectin-binding proteins involved in glycosylation processing pathways between highly and lowly metastatic NPC cells supported this notion. It is notable that several proteins which are recognized as metastasis-related molecules were differentially expressed in both NPC cell types. These include adhesion molecules such as L1 cell adhesion molecule (L1CAM), activated leukocyte cell adhesion molecule (ALCAM), major histocompatibility complex (MHC), CD44, CD166, integrin, vinculin, melanoma-associated antigen B2 (MAGE-B2), as well as other related proteins including mammalian ependymin-related protein 1 (MERP1), fibulin-7 (FBLN7), glial fibrillary acidic protein (GFAP), hornerin serpin H1 (SERPINH1), calumenin (CALU), aminopeptidase N (CD13), annexin A1 (ANXA1), peroxiredoxin 1, 2, and 4 (PRDX1, 2, 4), alpha-2-HS-glycoprotein (AHSG), cathepsin Z (CTSZ), protein S100 A8 and A9. Among the identified proteins, Galectin-3 was selected for further analysis of the protein levels and the extent of its possible role in NPC metastasis.

Galectin-3 belongs to a soluble glycoprotein binding family of immunoregulatory lectins, which exhibits an affinity for β-galactosides via a carbohydrate recognition that is either β-1,3 or β-1,4 linked to N-acetylglucosamine^21^. Although, thus far, there has been no experimental report whether Galectin-3 is glycosylated, however, based on artificial neural networks, the glycosylation has been predicted for this protein. It is also possible that Galectin-3 might bind to other glycoproteins, forming complex molecules, which could be pulled out together during the enrichment process. Galectin-3 can be found in both intracellular and extracellular compartments of the cells. The intracellular Galectin-3 can regulate cell proliferation, differentiation, survival, and cell death via many effector proteins such as K-Ras protein, Akt protein, and annexin VII, while the extracellular Galectin-3 mediates cell adhesion and cell signaling^22–24^. The released Galectin-3 can bind to components of extracellular matrix and its membrane counterparts of other cells, which have in a wide range of biological events including cellular homeostasis, immune function, angiogenesis, tumor invasion and metastasis^25^. Galectin-3 has been reported as a diagnostic or prognostic marker linked to metastasis in many types of cancer such as thyroid^26,27^, breast cancer^28^, melanoma^29^, lung cancer^30^, sarcoma^31^, gastric cancer^32^, prostate cancer^33^ and oral tongue cancer^34^. Here, we showed that Galectin-3 was greatly expressed in highly metastatic NPC cells compared to lowly metastatic cells. Furthermore, the downstream investigations including knockdown and overexpression of Galectin-3 in NPC cells as well as the treatment with specific inhibitor revealed that indeed galectin was involved in NPC cell metastatic phenotypes including adhesion, migration and invasion. For cell adhesion, a few studies demonstrated that overexpression of Galectin-3 enhance cell adhesion to ECM^35^. Our study showed that Galectin-3 rendered NPC cells to lose their ability to attach to ECM. These data have been linked to other investigations that various types of cancer cells which were incubated with Galectin-3 prior to plating, exhibited significantly reduced adhesion to ECM. It has been suggested that this phenomenon occurred as a result of interaction between Galectin-3 and alpha 1 beta 1 integrin^36^. Loss of cell adhesion allows cancer cells to escape from their site of origin and acquire a more motile phenotype. For migration and invasion, similar results have been observed in other cancers. Overexpression of Galectin-3 significantly increased migration and invasion whereas *gal-3* knockdown inhibited both processes in oral tongue squamous cell carcinoma^34^. Moreover, an siRNA against *gal-3* reduced migration and invasion in tongue cancer cell lines^37^. It has been proposed that Galectin-3 may regulate metastatic phenotypes via the Wnt/β-catenin signaling pathway^35,37^. Indeed, our data pointed to the possible involvement of the active β-catenin and potentially MAPK, AKT and NF-κB pathways. However, further studies are warranted to define the exact roles of these pathways in Galectin-3 mediated metastasis. In agreement with our findings, an immunohistochemical evaluation of 45 undifferentiated NPC tissues revealed that overexpression of Galectin-3 were independently correlated with poor overall survival^38^.

In summary, the current study provides clues for the involvement of a list of lectin-specific glycosylated proteins in NPC metastasis. The data from our findings will provide researchers more understanding about glycoproteins linked to metastasis and may help to develop targeted therapeutic drugs to reduce NPC progression. Galectin-3 has been shown to play a pivotal role in NPC metastasis in vitro. Further investigations including in vivo studies must be performed to determine whether Galectin-3 could be used for therapeutic intervention in human NPC metastasis.

## Methods

### Reagents and antibodies

RPMI 1640 media was purchased from GE Healthcare Hyclone (UT, USA). Antibiotics and fetal bovine serum (FBS) were purchased from Invitrogen (MA, USA). Anti-β-actin was purchased from Sigma-Aldrich (MO, USA). Anti-galectin-3 was purchased from Cell Signaling Technology (MA, USA). *Gal-3* siRNA, *Gal-3* expression plasmid, a scrambled sequence control and lipofectamine were purchased from Santa Cruz (TX, USA). Transwell chambers and Matrigel basement membrane matrix were obtained from Corning Life Sciences (MA, USA).

### Cell culture

Two human NPC cell lines 5-8F (highly metastatic) and 6-10B (lowly metastatic) derived from the parental line SUNE-1 were kindly obtained from Prof. Qingling Zhang, Southern Medical University, Guangzhou, China^39^. These cells were cultured in RPMI 1640, supplemented with 10% heat inactivated FBS and 100 U/ml penicillin and 100 µg/ml streptomycin, at 37 °C in a humidified 5% CO_2_ incubator. In certain experiments, cells were exposed to modified citrus pectin at the final concentration of 500 µg/ml for 24 h.

### Enrichment of lectin-specific glycoproteins in NPC cells

NPC cells (2 × 10^6^ cells) were plated in 10 cm^2^ culture dish for 48 h, and then were grown in serum free medium overnight. Cells and culture medium were collected at 72 h, lysed with specific lysis buffer and concentrated by centrifugation evaporator prior to the enrichment. Proteins (80 µg) were retrieved using lectin resin filled spin columns in Qproteome total glycoprotein kit (Qiagen, MD, USA) to capture ConA-enriched and WGA-enriched fractions. Briefly, the spin column was washed with 0.5 ml binding buffer containing 5 µl of protease inhibitor. Proteins were then loaded onto the spin column and incubated for 1 min. The unbound fraction on the column was removed by centrifugation at 100 × *g* for 2 min. The columns were washed twice with ice-cold binding buffer. The lectin binding proteins were then eluted, and the protein concentrations were measured by BCA method.

### SDS-PAGE and in-gel digestion

SDS-PAGE and in-gel digestion were performed as described previously with slight modifications^40^. Briefly, protein samples (30 µg) were resuspended in 4 × SDS sample buffer and subjected to 12% SDS-PAGE. SDS gels were stained with Coomassie Brilliant Blue G250, and destained in ultrapure water. For in-gel digestion, each gel lane was cut in pieces and destained in 50% acetonitrile in 50 mM NH_4_HCO_3_ until colorless and then newly prepared 10 mM dithiothreitol in 50 mM NH_4_HCO_3_ was added to reduce proteins at 60 °C for 15 min. Gel pieces were then cooled down to room temperature and newly prepared 55 mM iodoacetamide in 50 mM NH_4_HCO_3_ were added to alkylate the proteins for 30 min in the dark at room temperature. Thereupon, remaining solutions were removed and added with absolute acetonitrile to dehydrate the gel pieces. The gel pieces were let to completely dry. Trypsin enzyme solution (0.01 mg/ml) was directly added to digest the gel pieces and incubated at 37 °C overnight. To extract all peptides by directly adding 50% acetonitrile and eventually concentrated using an evaporator. The samples were kept at − 20 °C before to mass spectrometric analysis.

### Nano LC–MS/MS and data processing

Nano LC–MS/MS and data processing were performed as described previously with some modifications^41^. Briefly, all tryptic digested samples were resuspended in 0.1% formic acid and then analyzed by micrOTOF-Q II mass spectrometer (Bruker; Bremen, Germany) coupled with an UltiMate 3000 nano-LC system (Dionex; Surrey, UK). The separation flow rate was done at 300 nl/min. HPLC grade mobile phase A (0.1% (v/v) formic acid, 2% (v/v) acetonitrile in water) and HPLC grade mobile phase B (0.1% (v/v) formic acid in acetonitrile) were used to establish 50 min gradient. The gradient initiated with 10 min 2–10% B, followed by 33 min 10–40% B, ramped expeditiously (1 min) 40 – 95% B and maintained at 95% B for 1 min. The injection eluent was sprayed and ionized in the nano-electrospray source of mass spectrometer. Data were gained using Hystar software. The MS and MS/MS spectra were collected in mass range of m/z 400–2000 and m/z 50–1500, respectively. The data of mass spectrometric were converted into a mascot generic file (.mgf) using Mascot v.2.3.0 data analysis software version 4.0. (Matrix Science, London, UK) was used to discover data against SWISSPORT human protein database using trypsin enzyme with one possible missing cleavage allowed at 1 and the identified proteins were represented with more than one peptide. The precursor and fragment of mass tolerances ions were set to 1.2 Da and 0.6 Da, respectively. The peptide charge was chosen as 2+, 3+ and 4+ . Methionine oxidations and cysteine carbamidomethylation were set as variable modifications to reduce false positive identification. As a result of the same amount of proteins loaded into SDS-PAGE gel, semi-quantification information of 5-8F and 6-10B were achieved using exponentially modified protein abundance index (emPAI) provided by the Mascot. All protein identification lists were submitted predicted N-Glycosylation sites in human proteins using artificial neural networks using NetNGlyc 1.0 Server.

### Western blot analysis

Western blot analysis was performed as described previously with slight modifications^42^. Briefly, proteins from cell lysate were lysed with lysis buffer and centrifuged for 15 min at 12,000×*g* at 4 °C. The cell lysate supernatants were collected, and protein concentration was measured by Bradford assay. All samples from both cell lysates and culture media (~ 30 μg of protein/lane) were separated on 15% SDS-PAGE, and then transferred onto nitrocellulose membranes. Blots were blocked with 1% w/v bovine serum albumin (BSA) for 30 min at room temperature and were probed with anti-galectin-3 monoclonal antibody (1:500) and anti-β actin antibody (1:5,000) at 4 °C overnight. After incubation, the membranes were washed with phosphate saline buffer (PBS) supplemented with 0.1% Tween-20 and were incubated with corresponding secondary antibodies conjugated with horseradish peroxidase (1:1000) for 1 h. Finally, the immunoreactive bands were detected by chemiluminescence (Cell Signaling Technology).

### siRNA and plasmid transfection

Gal-3 siRNA was used in 5-8F cell transfection and Gal-3 expression plasmid was used to transfect 6-10B cells. Briefly, cells were seeded in 6 well plates and were incubated for 24 h. The cells were transfected with 40 nM of Gal-3 siRNA to 5-8F cells and 100 ng of Gal-3 expression plasmid to 6-10B cells using lipofectamine. Following 24 h incubation, culture medium was replaced, and the cells were further incubated for 48 h. Gal-3 expression in these cells were verified through Western blotting analysis.

### Cell proliferation assay

Cells were seeded in 96-well plates and subsequently cultured in growing medium. MTT (3-(4,5-dimethythiazol-2-yl)-2,5-diphenyl tetrazolium bromide) solution (0.5 mg/ml) was added to the cells after 24, 48, 72, 96, and 120 h and incubated for 3 h. Following the medium removal, 100 μl of 1:1 dimethyl sulfoxide and ethanol was added to each well to dissolve formazan crystals. The absorbance was measured at 540 nm using microplate reader. The proliferation rates were presented as a percentage of the control.

### Cell extracellular matrix adhesion assay

Cells supplemented in serum-free medium with 0.1% BSA were seeded in 24 well plates coated with Matrigel and were incubated for 2 h. Subsequently non-adherent cells were removed gently by washing twice with PBS and adherent cells were subjected to MTT assay. The absorbance of each well was measured at 540 nm. The adhesion index were presented as a relative value compared to the control.

### Migration assay

Cells were seeded into 24 well plates for 24 h to reach ~ 100% confluence. The wounds were created by scratching in each confluent monolayer using a pipette tip. Then cells were washed twice with PBS to remove cell debris. After 0 and 24 h, the images of cells were captured by a phase contrast microscope with a 10 × objective lens. The wound enclosure areas were determined using TScratch software to measure the distance traveled during the desired time^43^.

### Invasion assay

Invasion assay was performed using Transwell culture plates as described previously with slight adjustments ^44^. Briefly, chambers were pre-coated with Matrigel and culture medium for overnight. Cells were then seeded into the upper chamber in 0.5 ml culture medium with 1% FBS. Culture medium supplemented with 10% FBS was added in the lower chamber as a chemoattractant. After incubation for 24 and 48 h, cells migrated to the lower surface of the filters. The chambers were fixed in 0.1% paraformaldehyde followed by 4% paraformaldehyde in PBS for 30 min, and then stained with crystal violet. The invasive cells were expressed as the average number of cells per microscopic field, from at least five fields of view per filter.

### Experimental design and statistical rationale

A pool of proteins from three biological replicates of 5-8F and 6-10B cell lysates and culture medium was processed and subjected to mannose-rich and sialic acid-rich lectin columns. All the samples were processed with two technical replicates. For all other experiments, at least 3 independent replicates were performed, and the representative data are shown in Figures. Data were expressed as the mean ± standard error from at least 3 independent experiments. All data were considered significant at P values < 0.05. Statistical analysis was executed using SPSS 13.0 (SPSS, IL, USA) to compare between groups.

## Supplementary information

Supplementary Legend.

Supplementary Figure 1.

Supplementary Information.

## Data Availability

Raw mass spectrometric data were deposited to PeptideAtlas (Dataset Identifier: PASS01171).

## Abbreviations

ConA: Concanavalin A
Gal-3: Galectin-3
GeLC-MS/MS: Gel electrophoresis followed by liquid chromatography-tandem mass spectrometry
GlcNAc: N-Acetyl glucosamine
NPC: Nasopharyngeal carcinoma
WGA: Wheat germ agglutinin
