# Supplementary material for: Lectin affinity chromatography and quantitative proteomic analysis reveal that galectin-3 is associated with metastasis in nasopharyngeal carcinoma
Source: Sci Rep. 2020 Oct 5;10:16462. doi: 10.1038/s41598-020-73498-y (PMC7536187; doi:10.1038/s41598-020-73498-y)
Supplement: Supplementary file 1 — Supplementary Legend. [file 41598_2020_73498_MOESM1_ESM.docx]

**Supplementary Figure**

**Figure S1** The expression of signaling proteins that might be potentially involved in Galectin-3 mediated metastasis. The siRNA (siGal-3) and control siRNA (siControl) were transfected into 5-8F cells, while Galectin-3 expression (pGal3) and control (pControl) plasmids were transferred into 6-10B cells. The expression of indicated proteins was evaluated in cell lysates from NPC cells using Western immunoblotting with specific monoclonal antibody.
