# Supplementary figures and images for: Lectin affinity chromatography and quantitative proteomic analysis reveal that galectin-3 is associated with metastasis in nasopharyngeal carcinoma
Source: Sci Rep. 2020 Oct 5;10:16462. doi: 10.1038/s41598-020-73498-y (PMC7536187; doi:10.1038/s41598-020-73498-y)

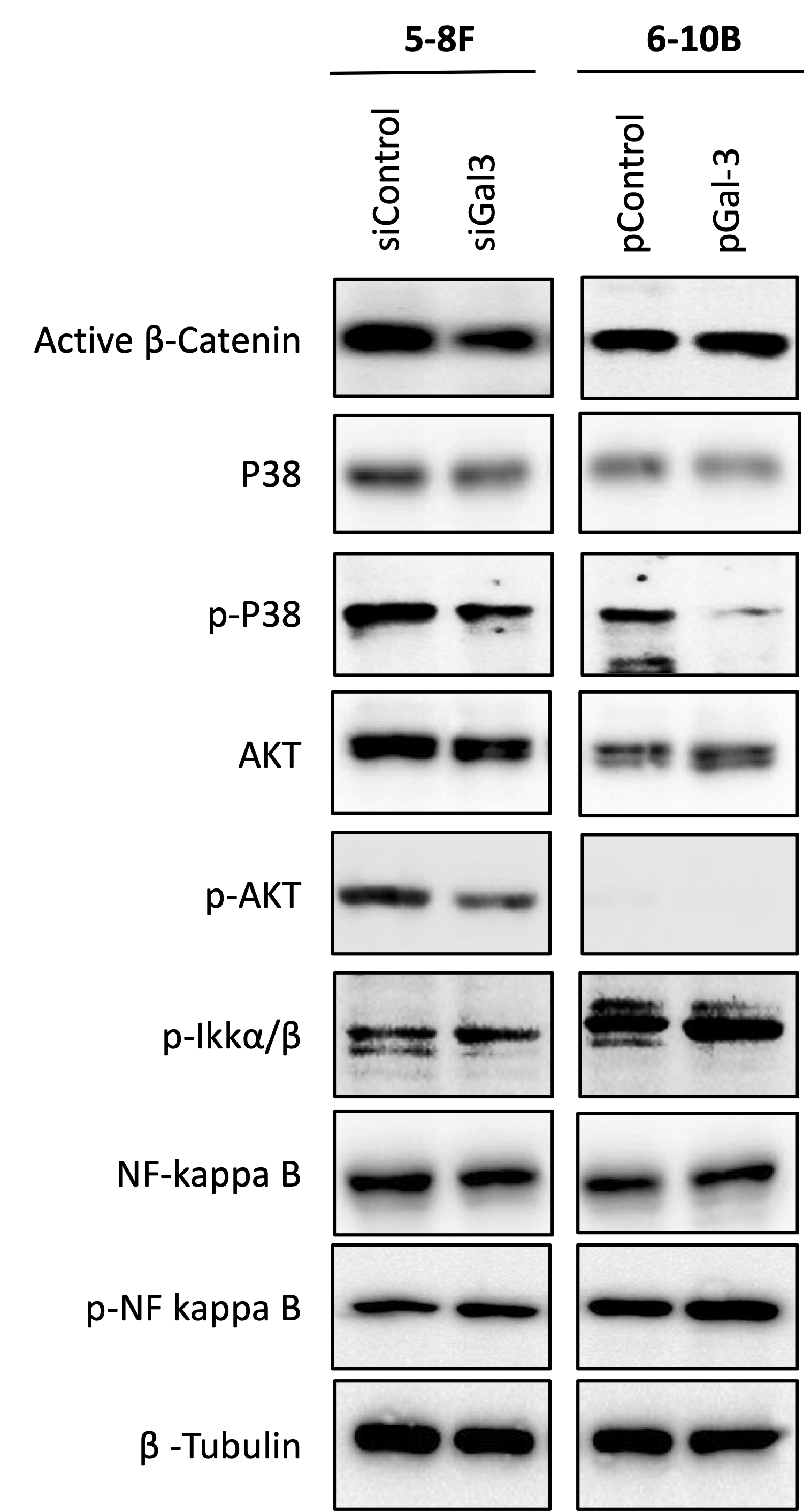

Supplement: Supplementary file 2 — Supplementary Figure 1. [file 41598_2020_73498_MOESM2_ESM.tiff]
