# Supplementary material for: Lectin affinity chromatography and quantitative proteomic analysis reveal that galectin-3 is associated with metastasis in nasopharyngeal carcinoma
Source: Sci Rep. 2020 Oct 5;10:16462. doi: 10.1038/s41598-020-73498-y (PMC7536187; doi:10.1038/s41598-020-73498-y)
Supplement: Supplementary file 3 — Supplementary Information. [file 41598_2020_73498_MOESM3_ESM.pptx]

## Slide 1
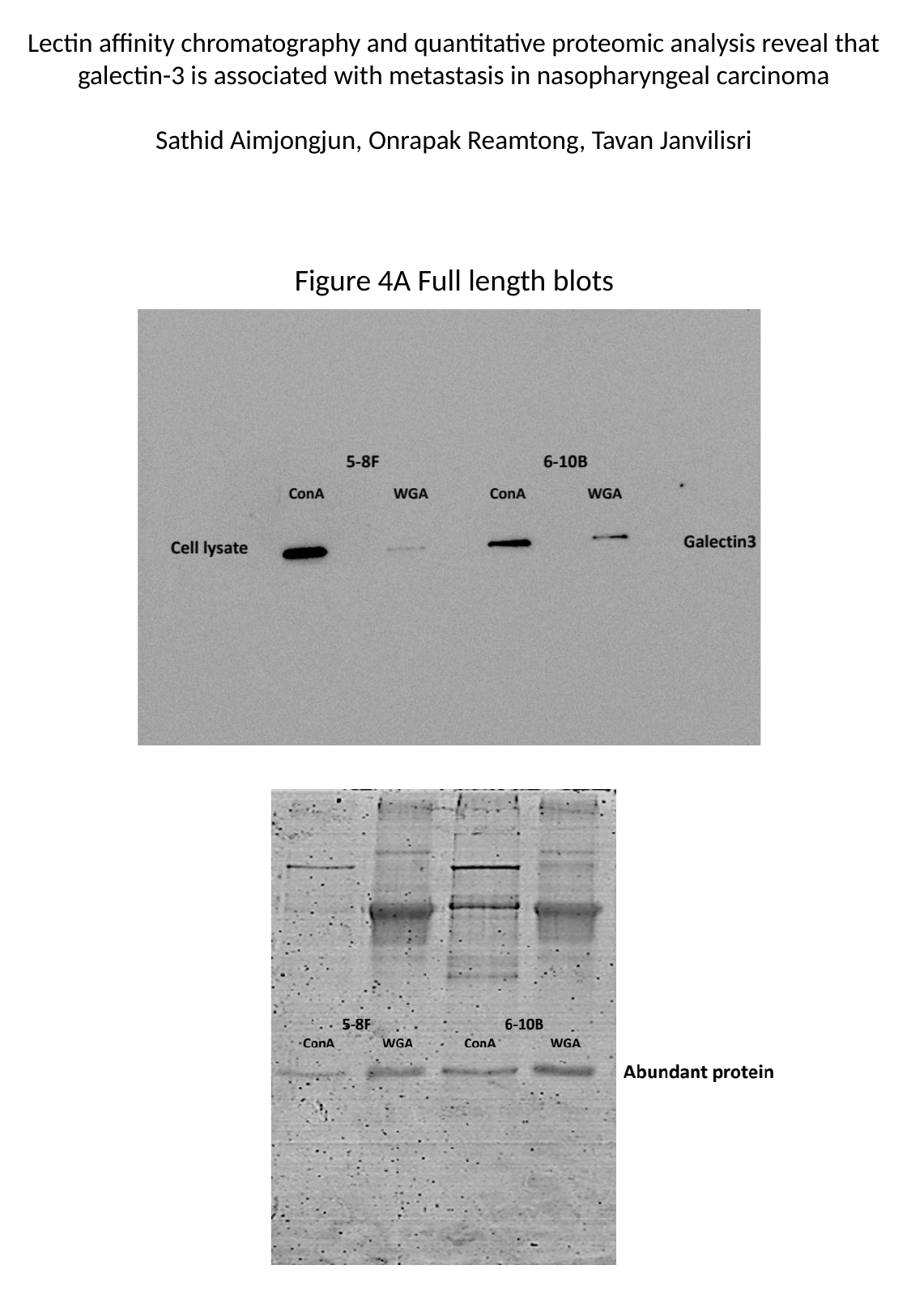

Lectin affinity chromatography and quantitative proteomic analysis reveal that galectin-3 is associated with metastasis in nasopharyngeal carcinoma
Sathid Aimjongjun, Onrapak Reamtong, Tavan Janvilisri
Figure 4A Full length blots

## Slide 2
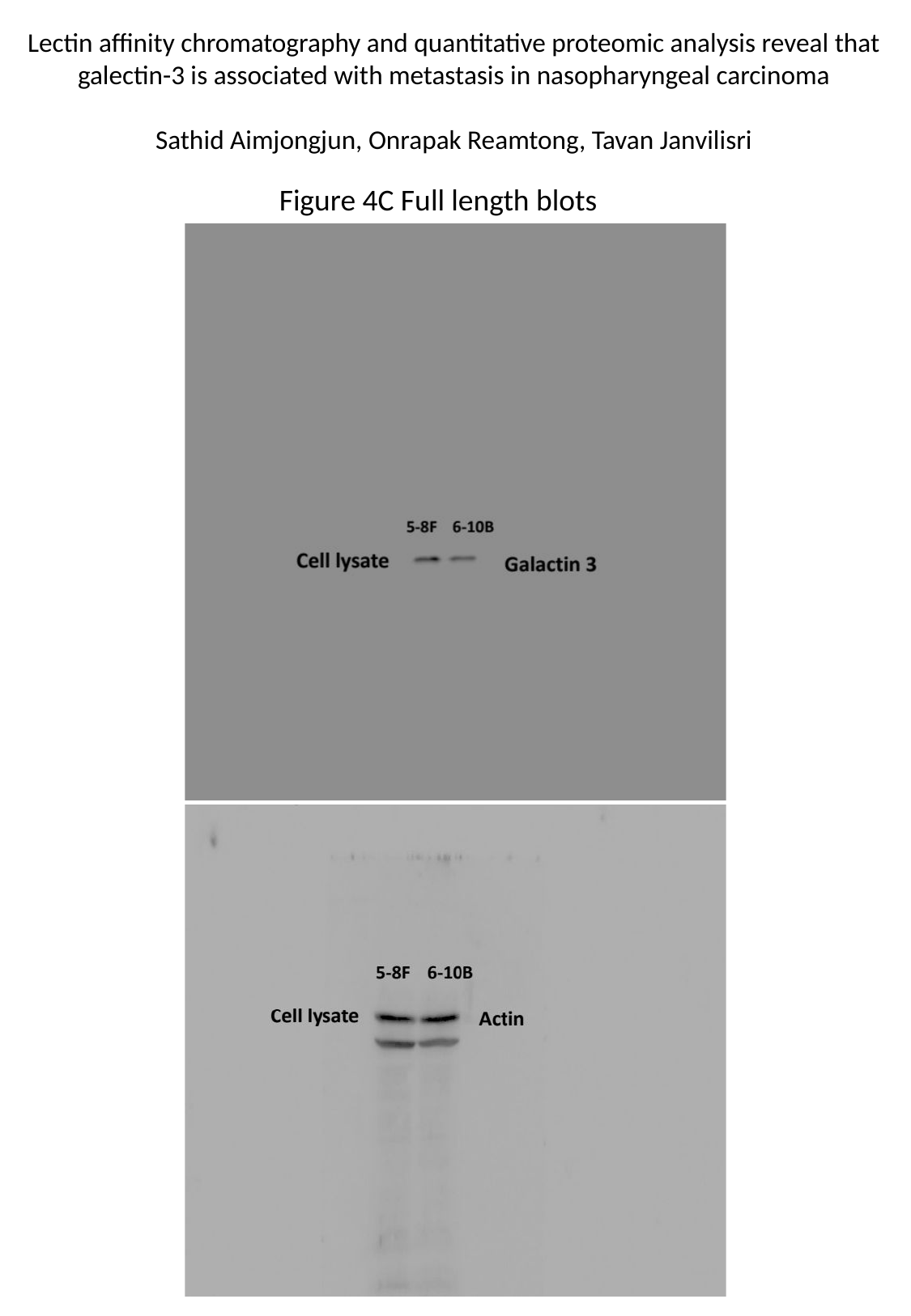

Lectin affinity chromatography and quantitative proteomic analysis reveal that galectin-3 is associated with metastasis in nasopharyngeal carcinoma
Sathid Aimjongjun, Onrapak Reamtong, Tavan Janvilisri
Figure 4C Full length blots

## Slide 3
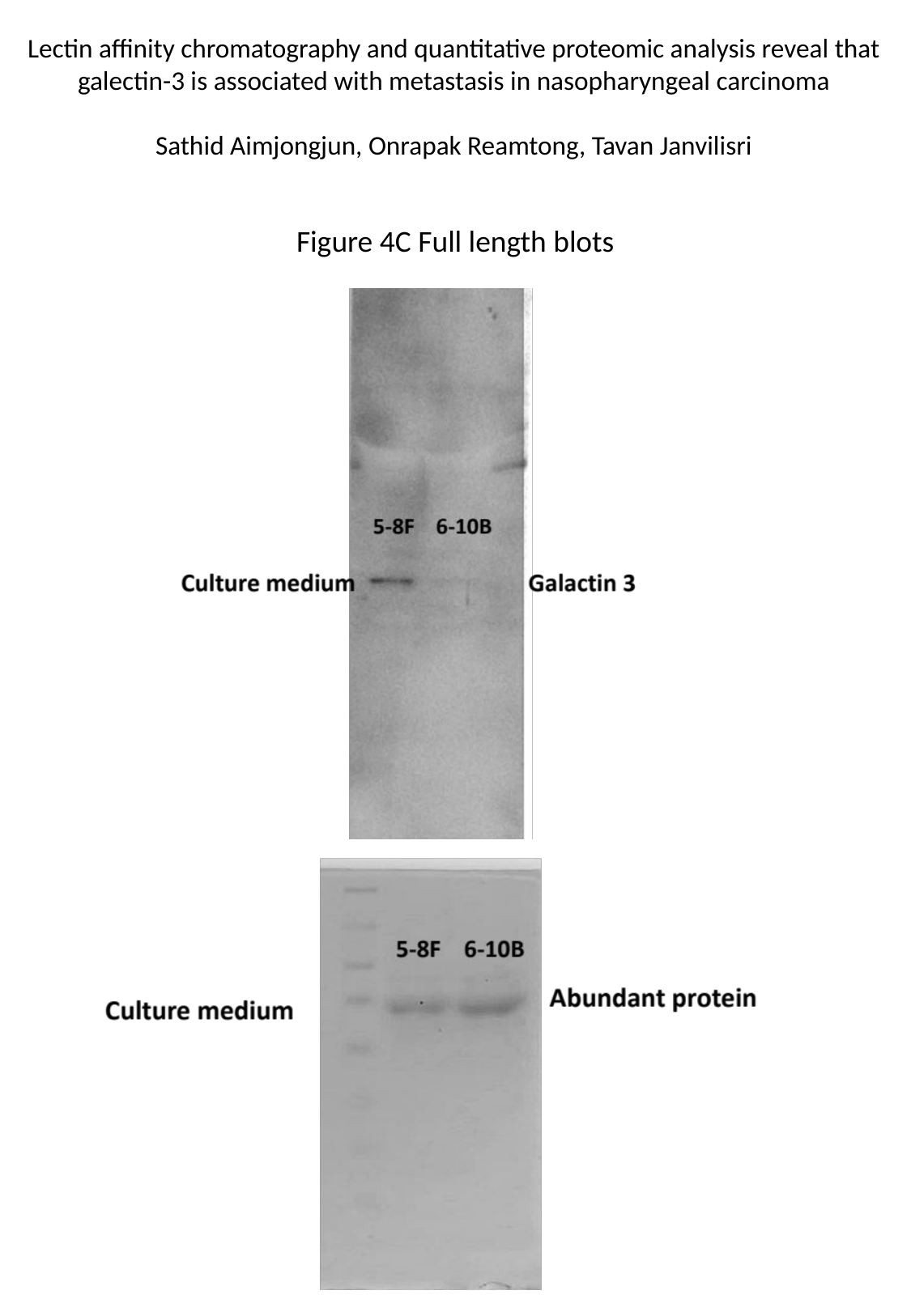

Lectin affinity chromatography and quantitative proteomic analysis reveal that galectin-3 is associated with metastasis in nasopharyngeal carcinoma
Sathid Aimjongjun, Onrapak Reamtong, Tavan Janvilisri
Figure 4C Full length blots

## Slide 4
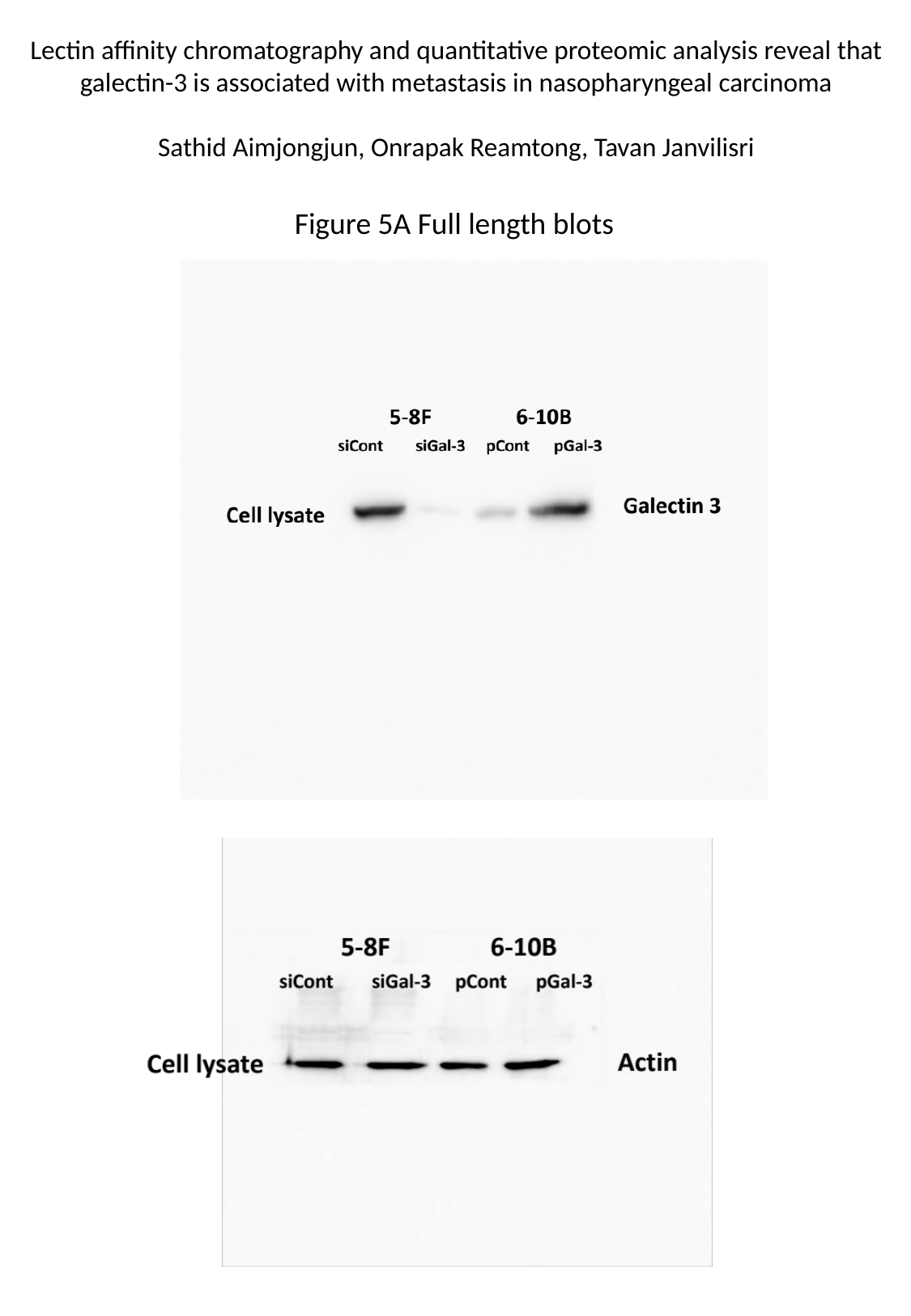

Lectin affinity chromatography and quantitative proteomic analysis reveal that galectin-3 is associated with metastasis in nasopharyngeal carcinoma
Sathid Aimjongjun, Onrapak Reamtong, Tavan Janvilisri
Figure 5A Full length blots

## Slide 5
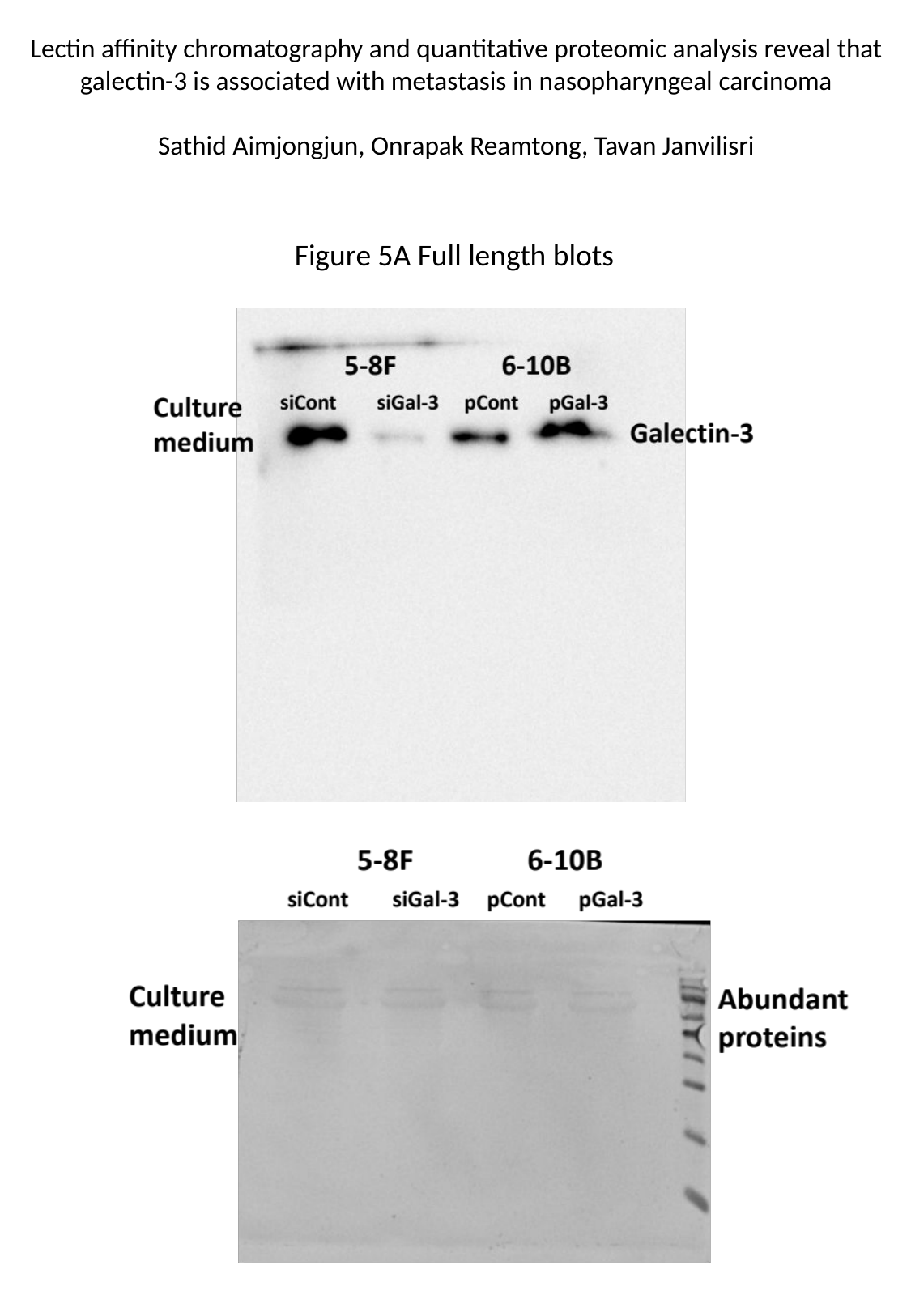

Lectin affinity chromatography and quantitative proteomic analysis reveal that galectin-3 is associated with metastasis in nasopharyngeal carcinoma
Sathid Aimjongjun, Onrapak Reamtong, Tavan Janvilisri
Figure 5A Full length blots

## Slide 6
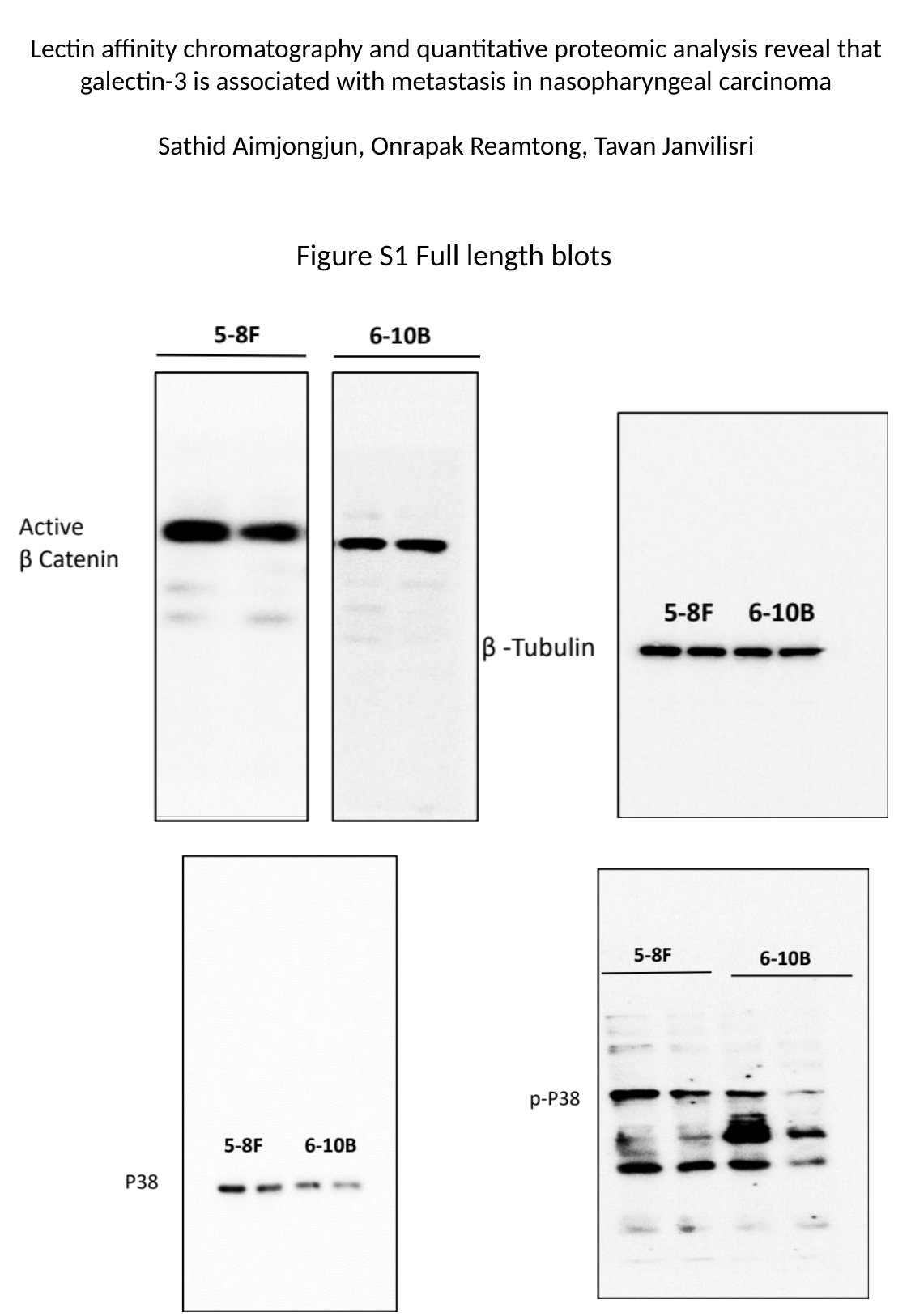

Lectin affinity chromatography and quantitative proteomic analysis reveal that galectin-3 is associated with metastasis in nasopharyngeal carcinoma
Sathid Aimjongjun, Onrapak Reamtong, Tavan Janvilisri
Figure S1 Full length blots

## Slide 7
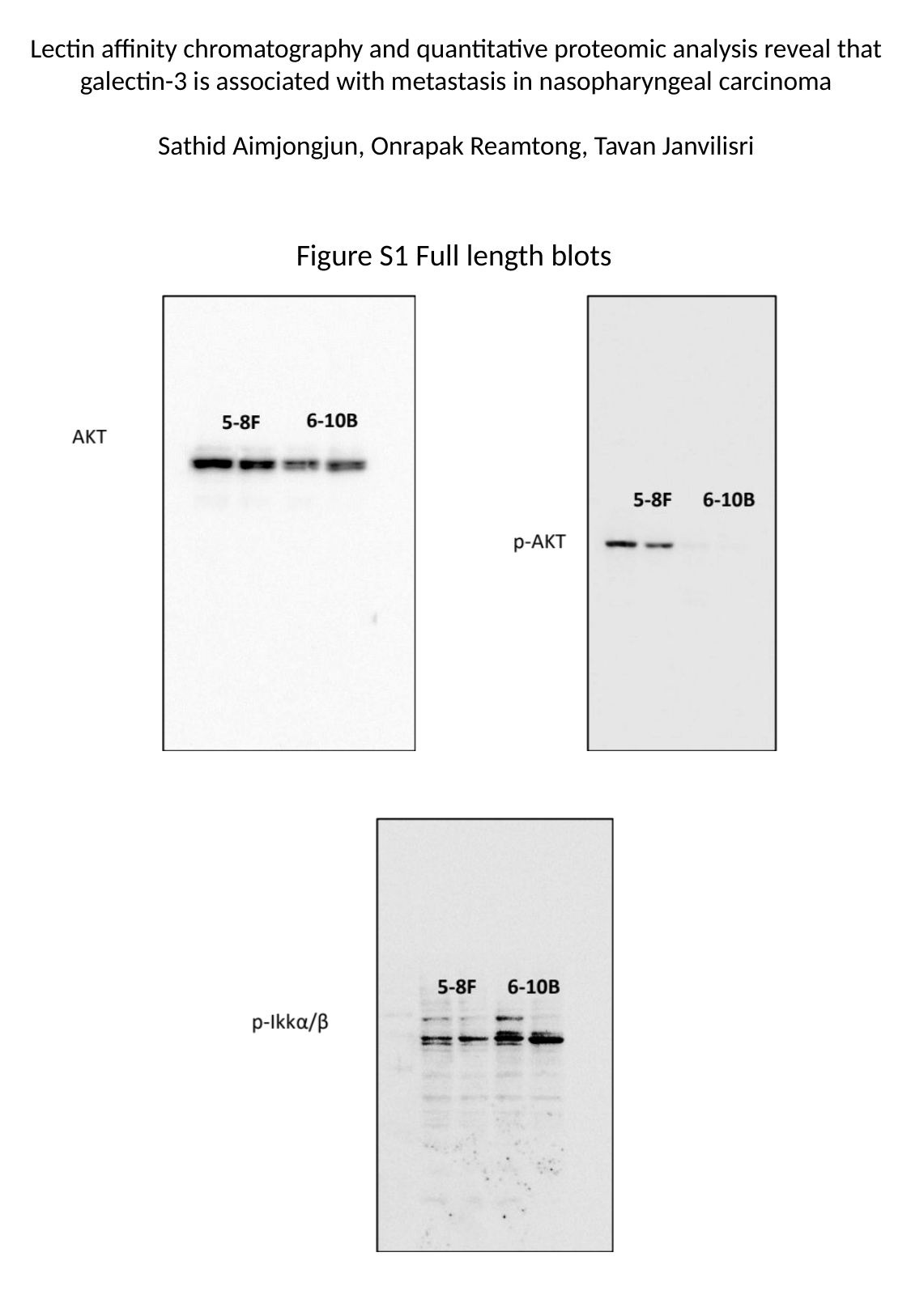

Lectin affinity chromatography and quantitative proteomic analysis reveal that galectin-3 is associated with metastasis in nasopharyngeal carcinoma
Sathid Aimjongjun, Onrapak Reamtong, Tavan Janvilisri
Figure S1 Full length blots

## Slide 8
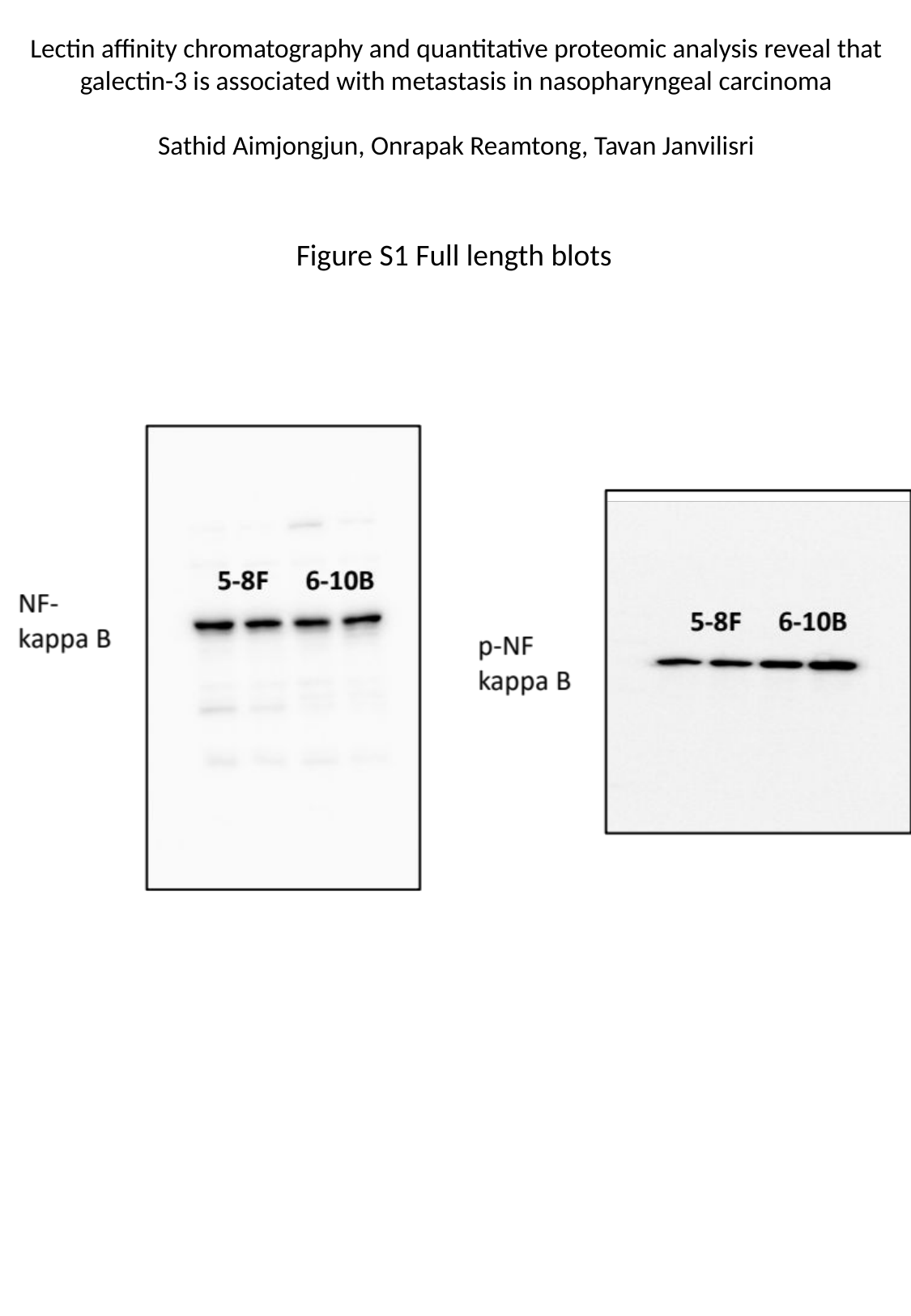

Lectin affinity chromatography and quantitative proteomic analysis reveal that galectin-3 is associated with metastasis in nasopharyngeal carcinoma
Sathid Aimjongjun, Onrapak Reamtong, Tavan Janvilisri
Figure S1 Full length blots
